# Individual Differences in Heartbeat‐Tone Synchronicity Judgments Suggest the Two‐Alternative Heartbeat Detection Task Is a Poor Test of Cardiac Interoceptive Accuracy and Insight

**DOI:** 10.1111/psyp.70310

**Published:** 2026-05-13

**Authors:** Kiera Louise Adams, Ren Palmer, Jonathan M. Bird, David Plans, Adam Cunningham, Davide Morelli, Tom Piercy, Ria Spooner, Rhea Clemente, Nerea Irigoras Izagirre, Elisa Fernandez Fueyo, Dawn Watling, Rebecca Brewer, Gemma Budworth, Dorina Cocirla, Mateo Leganes‐Fonteneau, Jennifer Todd, Jane E. Aspell, Geoffrey Bird, Jennifer Murphy

**Affiliations:** ^1^ Department of Experimental Psychology University of Oxford Oxford UK; ^2^ Olga Tennison Autism Research Centre, School of Psychology and Public Health La Trobe University Bundoora Australia; ^3^ Department of Psychology University of Surrey Surrey UK; ^4^ Department of Targeted Intervention University College London London UK; ^5^ Department of Psychology Royal Holloway, University of London Egham UK; ^6^ Huma Therapeutics Ltd London UK; ^7^ Department of Engineering Science University of Oxford Oxford UK; ^8^ Independent Scholar London UK; ^9^ Department of Biological Sciences Royal Holloway, University of London Egham UK; ^10^ Department of Anthropology University College London London UK; ^11^ Addiction Development and Psychopathology (ADAPT)‐Lab, Department of Psychology and Center for Urban Mental Health University of Amsterdam Amsterdam the Netherlands; ^12^ Louvain Experimental Psychopathology Research Group, Psychological Sciences Research Institute UCLouvain Louvain‐la‐Neuve Belgium; ^13^ School of Psychology and Sport Science Anglia Ruskin University Cambridge UK; ^14^ Centre for Research in Autism and Education, Institute of Education University College London UK; ^15^ European Research College London London UK; ^16^ European Research University Ostrava Czech Republic

**Keywords:** cardioception, HDT, heartbeat detection task, Interoception, interoceptive accuracy, interoceptive insight

## Abstract

Despite concerns regarding its validity, the two‐alternative forced‐choice heartbeat detection task (2AFC‐HDT) is a frequently used measure of cardiac interoceptive accuracy. In this task, participants must decide whether a series of tones occur synchronously with their heartbeats. One series of tones is predefined by the researcher as synchronous with heartbeats, and one series is predefined as asynchronous. The 2AFC‐HDT may result in individuals judged to be not interoceptive when they are, either if participants perceive their heartbeats as occurring synchronously with tones predefined as asynchronous rather than synchronous with their heartbeats, or if they do not perceive either set of tones as synchronous. Currently, there is little data on the proportion of participants this may affect. We addressed this using data from the Phase Adjustment Task (PAT) – a measure of cardiac interoceptive accuracy that determines if, and when in the cardiac cycle, a participant can perceive their heartbeat. The timing of heartbeat perception in 43 interoceptive participants was compared to the timing of synchronous and asynchronous tones used in the 2AFC‐HDT, assuming temporal precision of 50, 100, and 150 ms. Results suggest that between 53.5%–97.7% of delay‐based interoceptive individuals perceive heartbeats at a delay that does not correspond to the typical asynchronous or synchronous delays used to present tones on the 2AFC‐HDT. These issues suggest that the 2AFC‐HDT (or other measures that make assumptions about perceived timing of heartbeats) should not be used to measure cardiac interoceptive accuracy, or cardiac interoceptive insight (also known as awareness or metacognition).

## Impact Statement

This study shows that using fixed delays in the two‐alternative forced‐choice version of the heartbeat detection task for measuring cardiac interoceptive accuracy may result in false negatives. Interoceptive participants may perceive their heartbeats closer to the predefined asynchronous timing or as equidistant between synchronous and asynchronous conditions. This is the first study to demonstrate this effect in a large sample, suggesting that this task may not be suitable for measuring interoceptive accuracy or insight.

## 1 Introduction

Interest in interoception (the processing of one's own internal bodily signals; Craig 2002) is increasing due to its theorized role in numerous mental health conditions and aspects of higher‐order cognition (Brewer et al. 2021; Khalsa and Lapidus 2016). Unfortunately, there is growing concern regarding the validity of measures used to assess individual differences in interoception (Adams, Edwards, et al. 2022; Adams, Murphy, et al. 2022; Desmedt et al. 2025).

The most extensively studied facet of interoception is cardiac interoceptive accuracy^1^ – the ability to accurately perceive one's heartbeat. The Heartbeat Counting Task (Schandry 1981) has been the most popular test of cardiac interoceptive accuracy. In this task, participants are asked to count the number of heartbeats they perceive in a given time window, and their count is compared to an objective measurement to determine accuracy. However, its usage has decreased due to the impossibility of guarding against false positives (if participants know their heart rate, then they can appear interoceptive^2^ without being able to feel their heartbeats at all; Desmedt et al. 2018; Murphy et al. 2018; Brener and Ring 2016; Murphy 2024).

These concerns have prompted a renewed focus on Heartbeat Detection Tasks (first proposed by Whitehead et al. 1977; e.g., Dobrushina et al. 2022; McIntosh et al. 2022). In its simplest and most‐used form (Hickman et al. 2020; Adams, Edwards, et al. 2022; Adams, Murphy, et al. 2022), this task requires participants to determine whether a series of auditory or visual stimuli are in or out of sync with their heartbeats (the two‐alternative forced‐choice [2AFC] design). Researchers use a number of different methods to score this task (e.g., percentage accuracy, d prime, A prime), but all are designed to determine whether a participant can judge whether tones are synchronous with their heartbeats or not. Other variants present tones at multiple delays after cardiac contraction and assess the *consistency* of synchronicity judgments (e.g., the six‐alternative forced‐choice design [6AFC] and method of constant stimuli [MCS]; Brener et al. 1993; Clemens 1984; Yates et al. 1985). Scoring is either based on the interquartile range (where more distributed responses indicate a greater spread of synchronous judgments across presented intervals) or by a chi‐square analysis comparing responses to chance.

These three variants show only “adequate” convergent validity (Abma et al. 2016; Brener et al. 1993; Ring and Brener 2018). One study reported correlation coefficients of 0.72 between the 6AFC and MCS, −0.59 between the 2AFC and MCS, and − 0.55 between the 2AFC and 6AFC (the latter two negative correlations indicate that participants who are judged to be able to detect their heartbeats on the 2AFC are more consistent using the other two variants; Brener et al. 1993). These correlations are not as high as one might expect given the similar task demands of matching the timing of an external stimulus to one's heartbeat. Whilst the particular scoring approaches adopted may alter the size of the correlation between task scores, it is notable that the same study also demonstrated a discrepancy in the percentage of participants categorized as interoceptive across tasks using a binary scoring approach (where participants are classified as interoceptive or not), with 54% in the MCS, 50% in the 6AFC, and only 33% in the 2AFC.

Although it is possible that the MCS and 6AFC are overly liberal in classifying participants as interoceptive, a more plausible explanation for the lower‐than‐expected correspondences and the reduced proportion of interoceptive participants in the 2AFC‐HDT relates to assumptions about the timing of heartbeat perception in the 2AFC‐HDT. In the 2AFC‐HDT, researchers present tones that they expect to be perceived as synchronous with heartbeats approximately 200 ms after the r wave (the signature of myocardial electrical depolarization triggering ventricular contraction) and asynchronous stimuli approximately 500 ms after the r wave. In early instantiations of the task, delays of approximately 128 ms were chosen for synchronous tones based on the logic that this was how long it would take for the pulse to reach the neck, where participants were assumed to detect their heartbeats (Whitehead et al. 1976; Körmendi and Ferentzi 2024). This tone delay was also assumed to coincide with contraction of the heart, which was when several researchers assumed that individuals perceived their heartbeat (Whitehead et al. 1977; Schandry and Specht 1981; Katkin 1985). These tone delay choices were then supported by research using the MCS and 6AFC (Betka et al. 2020; Yates et al. 1985; Brener and Kluvitse 1988; Brener and Ring 1992; Brener et al. 1993), which show that *on average*, participants perceive tones presented at delays between 100 and 300 ms from the *r* wave as synchronous with their heartbeats.

In variants of the 2AFC‐HDT procedure that detect heartbeats using the arrival of the pulse wave at the finger instead of the r wave, delays are adjusted to account for the pulse arrival time, which is the time from the r wave to when the pulse is detected at the finger. These tasks typically use delays of 0 ms (from detection of pulse wave at the finger) as synchronous and 300 ms as asynchronous (Betka et al. 2018; Ewing et al. 2017), although in practice accounting for the pulse arrival time is difficult given the substantial individual variability in the pulse arrival time according to factors such as age and arm length (Budiman et al. 2022; see Wibmer et al. 2014; Chan et al. 2007, for variation in reported finger pulse arrival times).

Other variants of the 2AFC‐HDT present tones not after a fixed delay following the r wave, but instead at fixed proportions of the inter‐beat interval (the time between heartbeats; IBI; Knoll and Hodapp 1992; Lyyra and Parviainen 2018; Salomon et al. 2016; Mul et al. 2018). For example, in one study the asynchronous tones were presented at either 80% or 120% of the IBI (Salomon et al. 2016). These methods attempt to account for the fact that it might be the point in the cardiac cycle (e.g., systole vs. diastole) rather than the delay following the r wave, that determines when participants perceive their heartbeats. Whilst this may be appropriate for individuals who perceive heartbeat‐tone synchrony at a specific phase of the cardiac cycle (see also Palmer, Morelli, et al. under review), for those who perceive heartbeats at a specific delay, presentation of tones at specific cardiac phases under conditions of high heartrate variability will cause tones to be presented at different delays, meaning that ‘synchronous’ tones can no longer all be perceived as synchronous with heartbeats.

As detailed above, studies using MCS and 6AFC designs show *on average* individuals perceive stimuli presented at delays of approximately 200 ms as synchronous with their heartbeats. However, it is notable that there is a significant range in the delays producing synchronous judgments across individuals from 0 to 400 ms (Clemens 1984; Yates et al. 1985; Brener et al. 1993). This illustrates a problematic assumption of the 2AFC‐HDT: that the point within the cardiac cycle when participants perceive an external stimulus as synchronous with their heartbeat is the same for all individuals. Consider the following scenario: one interoceptive individual perceives external stimuli as synchronous with their heartbeat when presented 100–200 ms after the r wave, whereas another interoceptive individual perceives external stimuli as synchronous when presented 400–500 ms after the r wave. Although the delays at which they perceive synchrony differ, they can both perceive their heartbeats and so should both be determined to be interoceptive (Ring and Brener 2018). However, if participants are deemed interoceptive only if they consistently select the delay deemed as synchronous by the researcher (as is the case for most studies employing the 2AFC‐HDT task; e.g., Garfinkel et al. 2016; Herman et al. 2019), the 2AFC‐HDT would only categorize the former individual as interoceptive. As such, the 2AFC‐HDT is liable to false negatives (i.e., individuals who are truly interoceptive are determined not to be interoceptive). This may explain the lower‐than‐expected correspondence between the 2AFC and the MCS and 6AFC forms of the HDT, the reduced rate of individuals deemed interoceptive when assessed using the 2AFC‐HDT (Brener et al. 1993), as well as introduce noise into studies attempting to determine the relationship between cardiac interoceptive accuracy and any other variable of interest when cardiac interoceptive accuracy is measured using the 2AFC‐HDT.

The potential for false negatives would be reduced if participants who consistently judged either the predefined synchronous or asynchronous tone series to be synchronous were defined as interoceptive (i.e., if both positive and negative d prime or A prime values were used to indicate consistent selections; see Brener et al. 1993). Even if this practice was adopted, however, participants who perceive their heartbeats at delays other than those predefined as synchronous or asynchronous may still be judged as not interoceptive when they truly are, as they have no basis to consistently judge tones at either the predefined synchronous or asynchronous delays as synchronous.

There is little existing data to determine the perceived timing of heartbeats by interoceptive participants. Such data is crucial to determine the potential for false negatives in the 2AFC‐HDT. Data from an extremely limited number of participants suggests that individuals may perceive their heartbeats at a time that does not correspond to the synchronous condition in the 2AFC‐HDT. For example, Brener et al. (1993) found that six out of 24 participants who completed the 2AFC‐HDT, and one out of eight interoceptive participants, judged tones presented at the “asynchronous” *r* + 384 ms interval to be more synchronous with their heartbeats than the “synchronous” *r* + 128 ms interval. Wittkamp et al. (2018) used the 2AFC‐HDT and reported d’ values ranging from 4.48 (indicating a consistent preference for synchronous conditions) to −4.48 (indicating a consistent preference for asynchronous conditions). Collectively, these findings suggest that at least some participants perceive 2AFC‐HDT stimuli predefined as *asynchronous* to be *synchronous* with their heartbeats. By way of an illustration of this problem, Ring and Brener (2018) used data from the MCS task to plot the percentage of simultaneous judgments for each of the delays at which tones are presented in the task (0–500 ms at 100 ms intervals) for 13 interoceptive participants. This plot showed that although there was an overall tendency for tones presented at a delay of 200 ms to be most frequently judged as synchronous, there was a spread of delays selected by participants, from 100 to 400 ms. Despite the utility of these data in showing that false negatives in the 2AFC‐HDT are a practical problem, the available data is limited for several reasons; (1) the sample sizes are very small, making it difficult to adequately determine the scale of the false negative problem; (2) the delays used in the MCS do not span the entire cardiac cycle (unless a participants' heart rate is 120 bpm), and (3) analyzes examining the spread of synchronicity judgments are separated into 100 ms bins (which may over/underestimate the spread of responses if the range of perceived synchrony crosses the boundary of two bins; e.g., the MCS treats a perceived range of synchrony judgments between 299 and 301 ms as quantitatively the same as between 201 and 399 ms). Therefore, despite evidence that demonstrates that false negatives exist, existing data is insufficient to provide a reasonable estimate of their frequency.

Estimating the frequency of false negatives is made even harder due to inconsistency in test administration. Of particular relevance is the substantial variability in the delays used as synchronous in the literature. For example, synchronous presentations range from 0 (Hina and Aspell 2019; Mul et al. 2018; Lyyra and Parviainen 2018) to 300 ms following r wave detection (Gardner et al. 1990; Schirmer‐Mokwa et al. 2015). Similarly, asynchronous delays vary from 380 (Lombardo and Epstein 1986) to 600 ms (Schirmer‐Mokwa et al. 2015). This heterogeneity may result in differences in the proportion of false negatives across studies, difficulty combining data in meta‐analyzes, failures to replicate, and potential bias arising from systematic differences in the timing of heartbeat perception across groups, resulting in erroneous conclusions regarding group differences (or lack of) in cardiac interoceptive accuracy.

The potential issue of false negatives on the 2AFC HDT has yet to be examined in a large sample. Existing research is limited as the MCS does not examine perceptions of heartbeat tone synchrony across the entire cardiac cycle, and the use of 100 ms bins may both over and underestimate the spread of responses, as detailed above. Accordingly, the aim of the present study was to conduct a secondary analysis of a large corpus of recent work that has employed the Phase Adjustment Task (PAT; Plans et al. 2021), described as one of the most promising new measures of cardiac interoceptive accuracy (Desmedt et al. 2023). In the PAT, participants rotate a dial to change the temporal relationship between a series of tones and their heartbeats until they perceive the tones as synchronous with their heartbeats. This allows for the determination of whether an individual is interoceptive, and if so, when they perceive their heartbeats. Importantly, unlike many measures of cardiac interoceptive accuracy, the participant can present tones across the entire cardiac cycle, making this task ideal to examine individual differences in the timing of heartbeat perception (and therefore in heartbeat‐tone synchrony judgments). To examine whether the timing of heartbeat perception as revealed by the PAT corresponds to the typical delay choices on the 2AFC task, we restricted analyzes to those individuals who consistently perceived their heartbeat at a certain delay after objective heartbeat detection (i.e., interoceptive individuals who exhibited above chance levels of consistency of delay selection). After quantifying the timing of this interoceptive sample's heartbeat perception, one can assess whether the delays used to present tones on the 2AFC would be perceived as synchronous at varying levels of temporal discrimination. Based on prior results, we hypothesized that there would be a subset of interoceptive individuals who would likely be falsely classified as non‐interoceptive on the 2AFC‐HDT, as they select delays that do not correspond to the synchronous (or asynchronous) condition of the 2AFC‐HDT. However, we made no predictions about the proportion of individuals affected by this issue, given the relatively small sample sizes employed by previous studies.

## 2 Methods

### 2.1 Participants

Data was pooled from previous studies (Todd et al. 2024; Spooner, Bird, Clemente, et al. 2024; Spooner, Bird, Irigoras Izagirre, et al. 2024) and unpublished work (see details in the Supporting Information S1). Although there were slight variations in the inclusion and exclusion criteria across studies, broadly, participants were over the age of 18 and spoke English fluently. After initial data cleaning to remove inattentive or unengaged respondents (those with four or fewer heart rate values indicative of poor engagement, or those containing 0 responses) and those with too few trials (< 17). Whilst previous work suggests 15–20 trials provides a reasonable trade‐off between task length and accuracy (Plans et al. 2021; Todd et al. 2024; Palmer, Murphy et al. under review), we selected 17 trials as the cutoff as this was the threshold pre‐registered by the majority of studies included (Spooner, Bird, Irigoras Izagirre, et al. 2024; Spooner, Bird, Irigoras Izagirre et al. 2024). If participants had completed the PAT on more than one occasion, due to taking part in several studies or longitudinal studies, the first valid completion was retained. This resulted in the removal of 74 datasets. The total sample size before only the interoceptive participants were selected was *N* = 381. In this total sample, *N* = 55 were interoceptive overall (14%) and *N* = 43 were included in the final sample as they exhibited a consistent delay‐based preference (rather than a preference for a specific phase of the cardiac cycle) for comparison with the 2AFC‐HDT intervals (see scoring; Table 1). Please see the Supporting Information an overview of the inclusion and exclusion criteria within each study, details of ethical approval, pre‐registrations and recruitment methods (Supporting Information S1), and for demographic information of the participants included in analyzes from each study (before only the interoceptive participants were selected; Supporting Information S2).

**TABLE 1:** Demographic information for interoceptive participants.

Sex (%)	Age in years (average/SD)	Presence of mental health condition (%)	Presence of physical health condition (%)
Male: 20.9%	29.1 (11.1)	79.1% no	44.2% no
Female: 76.7%		9.3% yes	2.3% yes
Not recorded: 2.3% *		11.6% not recorded	53.5% not recorded

*Note:* This table reflects the demographic information of the 43 participants across studies who were deemed to be interoceptive with a delay preference (see scoring). * 9 (20.9%) of these participants reported gender rather than sex. Demographic data were missing for 1 participant.

### 2.2 The PAT

The PAT was selected as unlike the MCS and 6AFC, the PAT enables the assessment of perceived heartbeat timing across the entire cardiac cycle for all participants. Although a relatively new measure, the PAT predicts scores on other interoceptive tasks but not matched exteroceptive tasks (Plans, Murphy, et al. under review), does not vary depending on remote vs. laboratory‐based administration (Palmer, Murphy, et al. under review), quantifies both phase‐ and delay‐based response patterns (Palmer, Murphy, et al. under review), and does not exhibit practice effects (Palmer, Spooner, et al. under review). However, it should be recognized that for the purposes of this study, the PAT needs to fulfill two criteria: (1) it is able to determine if participants can perceive their heartbeat; and (2) participants need to be able to indicate when they feel their heartbeats. With regard to the first criterion, as far as we are aware (like the 2‐ and 6‐AFC HDT tasks, and the MCS task), there has never been a claim that anything other than heartbeat perception can result in above chance performance on the PAT (unlike the HCT, where above chance performance can be achieved with heart rate knowledge and good duration estimation; Desmedt et al. 2023). With regard to the second criterion, the whole basis of the PAT is that it allows participants to indicate when they feel their heartbeats.

A smartphone application was used to administer the PAT. This application used a heartbeat detection algorithm developed in swiftUI by BioBeats Ltd. (Cropley et al. 2017; Morelli et al. 2018). Participants place their index finger over the camera lens and flash and photoplethysmography is used to detect the change in color at the finger as the participant's pulse wave arrives.

Participants are instructed to sit somewhere quiet, with the volume of their phone maximized and with “do not disturb” mode enabled. Subsequently, participants are required to watch a short video depicting the task instructions (see Supporting Information S3). Participants are instructed to hold their phone with one hand, with their index finger over both the camera lens and the flash. If participants move their finger, an on‐screen prompt is displayed asking them to reposition their finger before recording continues.

During each trial participants listen to a series of tones (with a duration of 156 ms) at the same frequency as their heart rate. In the original version of the PAT, smartphone application tones were triggered by the predicted occurrence of heartbeats derived from beat‐to‐beat intervals recorded every three seconds (Plans et al. 2021). In this updated version, tones were triggered by the detection of participants' heartbeats by the application (Palmer, Murphy, et al. under review). The starting delay (with respect to heartbeat detection by the application) for tones is random across trials. The random delay was generated using the standard pseudo random number generator in Swift. This scalar scales the delay using 0.5 multiplied by the greater of either the current—or trial average—IBI to produce the delay value. This produces an initial delay between 0 and the greater of half the current or trial averaged IBI. In addition, a second random number rotates the 0 point on the dial a number of radians between ‐Pi and Pi at the start of the trial. The application pauses presentation of tones if heartbeat detection is disrupted due to finger movements, and then restarts when there is a clear heartbeat signal. Participants are asked to rotate a virtual dial to advance or retard the tones in time until they perceive the tones to be synchronous with their heartbeats. Each turn of the dial advances or delays the tones by a set proportion of the time between heartbeats. This is to ensure that those with slower heart rates do not have to work harder to make the tones synchronous with their perceived heartbeats. Participants were free to make as many adjustments to the dial as they wished and pressed a button to confirm when they perceived the tones as synchronous with their heartbeat. Participants complete two practice trials, followed by 20 main task trials.

### 2.3 Scoring

#### 2.3.1 Cardiac Interoceptive Accuracy: Phase Adjustment Task

Scoring was performed on the first 17 trials of the PAT (excluding the practice trials), to ensure the same number of trials were included for each participant (as calculations are dependent on the number of trials). We used the updated scoring system for the PAT (Palmer, Murphy, et al. under review). In this revised approach, consistency is defined using delay values as periodic functions of the heartbeat period, calculated as: $\text{consistency}\;\left(d,p\right)=\frac{1}{n}\mathrm{mod}\left(\sum_{j=1}^{n}e^{i2\pi \frac{d_{j}}{p_{j}}}\right)$, where *d* is the delay and *p* is the heartbeat period.

Cardiac interoceptive accuracy is determined by two complementary scoring frameworks. Phase‐based consistency scoring captures individuals who consistently select the same phase of their cardiac cycle over trials, whilst delay‐based consistency scoring captures individuals who consistently select the same delays over trials regardless of their heartbeat period (Palmer, Murphy, et al. under review).

##### 2.3.1.1 Phase‐Based

To calculate phase‐based consistency, we expressed delays as phase angles relative to each trial's median IBI at the final dial position (*p*). In other words, delays are interpreted as a proportion of the trial IBI. To determine whether performance is above chance, a group‐level cutoff was derived from 100,000 simulations of random delay sequences and randomly sampled heartbeat periods (0.5–1.5 s). The 95th percentile of this null distribution serves as the threshold for classifying responses as non‐random (i.e., interoceptive).

##### 2.3.1.2 Delay‐Based

To calculate delay‐based consistency, fixed‐period scoring was utilized. Here, all delays are interpreted as an absolute value using a constant, participant‐specific period *p*, calculated as the participant's overall median of the final dial position median IBIs. Because individual variability in IBI affects the null distribution under this model (see Palmer, Murphy et al. under review), participant‐specific thresholds are generated from 10,000 simulated delay sequences per participant. The 95th percentile of each individual's null distribution is used as the cutoff for classifying their responses as interoceptive.

##### 2.3.1.3 Classification

Participants are classified as interoceptive if they score above the chance threshold under either scoring method, resulting in a binary (interoceptive vs. non‐interoceptive) score. Those classified as interoceptive under both methods were classified as delay‐based responders for the purpose of this analysis. This is because the 2AFC‐HDT focuses on selected delays and classification as interoceptive under both phase and delay scoring occurs with stable heart rates that render phase‐ and delay‐based responses indistinguishable (Palmer, Murphy, et al. under review). After excluding those who exhibited phase‐based response patterns (*N* = 12), the final sample size was 43 participants.

### 2.4 Analysis

#### 2.4.1 Timing of Heartbeat Perception (Perception of Heartbeat‐Tone Synchrony): Phase Adjustment Task

The delay at which each interoceptive individual perceived heartbeat‐tone synchrony on the PAT was calculated using circular averaging: each trial's delay selection was converted to a phase angle (in radians) using the participant's final sequence median IBI during over trials and averaged. The final median sequence IBI was calculated from the time the participant moved within 5° of their final selected angle to when they pressed confirm. The number of heartbeats within this time ranged from 1 to 128, with an average of 8.9 (SD = 9.6). Notably, we also considered using the final IBI of each trial for this calculation, but as the correlation between these two IBI values was very high (the correlation between the final value on each trial and the final sequence median IBI during the trial was *r* = 0.99; see Supporting Information S4), we used only the median of final sequence median IBIs. After conversion to a phase angle, these values were then transformed into Cartesian coordinates. For each participant, the mean vector was computed by summing the x and y components across trials, and the selected angle was derived using the arctangent of the summed coordinates. In practice this produces two delays (as heartbeats are cyclical—a positive delay from the preceding heartbeat and a negative delay from the following). In all analyzes we used the most conservative of these options (i.e., we used the delay closest to the synchronous, then the asynchronous tone, instead of a delay that was close to neither) to provide the most conservative estimate of interoceptive participants who would be misclassified using the 2AFC‐HDT.

#### 2.4.2 Comparing Delays at Which Tones Are Judged To Be Synchronous With Heartbeats to Delays Used in 2AFC‐HDT Studies

After quantifying the delay at which each interoceptive participant perceived heartbeat‐tone synchrony on the PAT, we compared these delays to those typically used to present ‘synchronous’ tones on the 2AFC‐HDT. This allows estimation of the proportion of interoceptive participants who would perceive tones presented at the standard synchronous 2AFC‐HDT delays as synchronous, and how many would not. As the PAT uses the arrival of the pulse wave at the finger to trigger tones when implemented as a smartphone application rather than the r wave from an electrocardiogram (ECG), we considered delays used in 2AFC‐HDT studies using the arrival of the pulse at the finger to present tones at different delays. We found seven studies that recorded the arrival of the pulse at the finger and presented tones following fixed delays from this event in the asynchronous and synchronous conditions (Betka et al. 2018; Ewing et al. 2017; Hart et al. 2013; Kandasamy et al. 2016; Leganes‐Fonteneau et al. 2019; Palser et al. 2018; Herman et al. 2019). In all studies, synchronous tones were defined as those occurring 0 ms after the arrival of the pulse at the finger, and asynchronous tones were defined as those occurring after a 300 ms delay. We therefore used 0 ms as our synchronous tone delay and 300 ms as our asynchronous tone delay.

In order to establish whether participants' selected delays align with the predefined synchronous tone series in the 2AFC‐HDT, we calculated the proportion of interoceptive participants' selected delays which fall within a temporal window centered on 0 ms delay (and on 300 ms for the ‘asynchronous’ tone series). The size of the temporal window reflects the assumed degree of perceptual temporal acuity (where if two stimuli occur within this window the participant would perceive them as occurring synchronously). There is not much information on the temporal precision with which individuals can perceive heartbeats, however, there is research suggesting the specificity of discrimination on the 6AFC (SD of the interval perceived to be synchronous) ranges from 43 to 167 ms (Brener and Kluvitse 1988). This suggests that within an approximately 100 ms window, participants may be unable to differentiate delays. To provide a comprehensive analysis, we conducted analyzes using temporal windows of +/− (a) 50, (b) 100, and (c) 150 ms around 0 and 300 ms.^3^ Participants can therefore be classified according to whether their timing of heartbeat perception aligns with the predefined synchronous tone on the 2AFC‐HDT, the asynchronous tone, or neither.

## 3 Results

**FIGURE 1:**
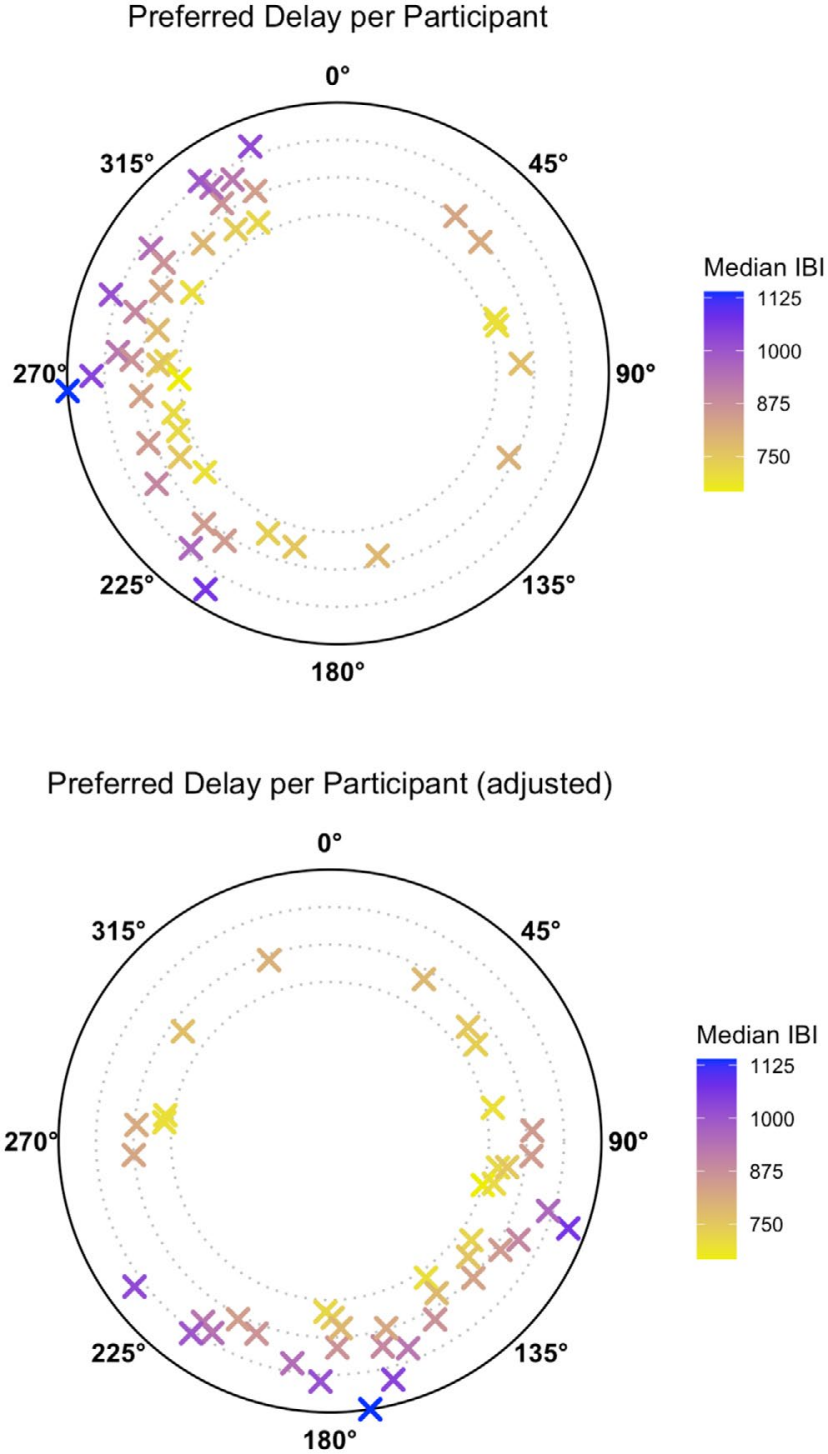
Delays selected by participants. Delays at which individuals perceived heartbeat‐tone synchrony for *N* = 43 interoceptive participants classified as delay‐based responders. Each participant is represented with a cross. Delays are represented as angles on IBI‐scaled circles (smallest to largest circles represent the smallest to largest IBIs; IBIs are represented in ms). The same data are presented twice but recalibrated such that 0° represents a delay of 0 (above) and 300 ms (below).

Figure 1 presents tone delay selections across all delay‐based interoceptive participants (individual participant trial‐by‐trial response data are provided in the Supporting Information S5). As can be seen, tone delay selections did not cluster at 0 ms, which is when the predefined synchronous tones are presented in the 2AFC‐HDT, and there was also not a cluster at the predefined asynchronous tone delay of 300 ms.

Table 2 presents the comparison between tone delays selected on the PAT by interoceptive participants with consistent tone delay selections, and delays defined as synchronous and asynchronous in 2AFC‐HDT studies where heartbeats are recorded at the finger. Three comparisons are presented, one for each of the three temporal windows assuming different degrees of perceptual temporal acuity. As can be seen, depending on the assumed temporal acuity, only 0%–32.6% of delay‐based interoceptive participants selected delays on the PAT that correspond to synchronous delays used in the 2AFC‐HDT. Furthermore, between 2.3% and 14.0% of participants perceived tones to be synchronous at delays thought to be asynchronous on the 2AFC‐HDT.

**TABLE 2:** The number of participants who selected tone delays aligned with the predefined synchronous and asynchronous delays used in the 2AFC‐HDT.

	Assumed temporal precision		
	50 ms	100 ms	150 ms
Synchronous (0 ms)	0 (0%)	9 (20.9%)	14 (32.6%)
Asynchronous (300 ms)	1 (2.3%)	5 (11.6%)	6 (14.0%)
Outside of window	42 (97.7%)	29 (67.4%)	23 (53.5%)

For these results in only participants who passed an exteroceptive screening task matched to the PAT in terms of demands, please see the Supporting Information S6.

## 4 Discussion

The aim of this study was to estimate the extent to which the timing of heartbeat perception corresponds to the typical delays used to present “synchronous” tones on the 2AFC, and therefore the likelihood of false negatives on the 2AFC‐HDT (individuals deemed not to be interoceptive when they in fact are). Tone delay selections from interoceptive participants who exhibited consistent tone delay selections on the PAT were compared with the predefined synchronous and asynchronous tone delays on the 2AFC‐HDT. Comparisons were conducted within three temporal windows, guided by Brener and Kluvitse (1988), since there is little empirical evidence on how far tones must be delayed from the perceived heartbeat before perception of the timing of the two events changes from synchronous to asynchronous. Across these temporal windows, only 0%–32.6% of delay‐based interoceptive participants perceived heartbeat‐tone synchrony at delays that correspond to those typically used to present synchronous stimuli on the 2AFC‐HDT. Similar results were found when restricting analyzes only to those participants who had also passed an exteroceptive screener task. Given that participants on the 2AFC‐HDT are asked to indicate if the ‘synchronous’ tones are synchronous with their perceived heartbeats, such a pattern is consistent with a high risk of false negatives on the 2AFC HDT, as previously indicated by comparison with other tasks such as the MCS (see Brener and Ring 2016).

As detailed in the Introduction, if d′ or A′ is used to score the 2AFC‐HDT, and negative values beyond a critical value taken as evidence of cardiac interoceptive accuracy, participants who consistently perceive tones at the ‘asynchronous' delay as synchronous with their heartbeat should be deemed to be interoceptive (see Brener and Ring 2016). If this scoring and classification approach were adopted, the current data suggests the percentage of false negatives on the 2AFC HDT would be decreased. Across the three temporal windows, between 2.3%–14.0% of delay‐based interoceptive participants perceived heartbeats at delays that correspond to those typically used to present asynchronous tones on the 2AFC‐HDT. As a consequence, these data suggest that between 53.5% to 97.7% of delay‐based interoceptive individuals perceive heartbeats at a delay that does not correspond to the asynchronous or synchronous delays used to present tones on the 2AFC‐HDT.

Critically, we do not know how the interoceptive individuals who perceive heartbeats at a time other than that judged to be synchronous with heartbeats on the 2AFC‐HDT respond on the 2AFC‐HDT. On each trial of the 2AFC‐HDT participants are presented with a series of tones and asked to make a binary judgment regarding whether the tones were synchronous or asynchronous with their heartbeat. If they strictly follow instructions, they should respond asynchronous each time and would be falsely judged to be non‐interoceptive. Other patterns of responding are possible of course, they may respond at random, they may learn across trials that tones occur at two delays and begin to respond ‘synchronous’ for the tone series that is closest to their perceived heartbeats. Depending on when (i.e., after how many trials) they make this realization and adopt this strategy, it is possible they may appear interoceptive. If participants have poor temporal acuity (i.e., a large temporal window) and perceive heartbeats at a point between the two delays used to present tones on the 2AFC‐HDT then tones presented at both delays may produce synchronous judgments. These possible patterns of responding may vary depending on the instructions used (e.g., that tones may only follow heartbeats, as used by a small number of studies; Kandasamy et al. 2016), and expectations regarding the proportion of synchronous and asynchronous presentations which may produce response biases. It is this uncertainty and likelihood of false negatives that means the 2AFC‐HDT is a poor test of cardiac interoceptive accuracy.

As the PAT measures the arrival of the pulse at the finger, we used the delays from studies which instantiate the 2AFC‐HDT using detection of the pulse at the finger. This decision was made given the huge variability in (and therefore difficulty of estimating) the pulse arrival time to the finger. As most 2AFC‐HDT studies measure heartbeats using the r wave, it could be argued that the issue illustrated here may not apply to all versions of the 2AFC‐HDT. In addition, it could be argued that these results do not apply to studies which present tones at set proportions of the participant's IBI. However, consistent with other studies that have examined both delay and phase‐based response patterns (e.g., Palmer, Morelli, et al. under review), tone delays selected as synchronous by interoceptive participants were distributed across the cardiac cycle. Taken together, these results and the results of other studies suggest that false negatives are likely to occur when using any instantiation of the 2AFC‐HDT. Importantly, the varying frequencies of participants' perception of heartbeats across the cardiac cycle also illustrates the issue with the variability of tone delays that are defined as synchronous and asynchronous in previous studies using the 2AFC‐HDT. The interaction between the choice of delays used in particular studies, and the specific delays preferred by each sample of participants in each study, would influence the proportion of false negatives and the specific participants who are deemed interoceptive versus non‐interoceptive. As such, the variability in tone delays used, and of individual differences in the timing at which different participants perceive heartbeats, impedes comparison across studies using the 2AFC‐HDT.

Although the 2AFC‐HDT and PAT were not directly compared, findings are broadly consistent with previous findings employing such approaches, with varying tone delays, in smaller samples. Brener et al. (1993) found that of 8 interoceptive participants who completed the 2AFC‐HDT, one perceived tones at the “asynchronous” (384 ms from r wave) tone delay as more synchronous than the “synchronous” (128 ms from r wave). Results also replicate the spread of tone delays selected by interoceptive participants shown by Ring and Brener (2018), but in a larger sample, and in a more fine‐grained manner that spans the entire cardiac cycle. Therefore, despite differences in analytic approaches and methods of measuring heartbeats, the results of these studies are consistent. The presence of false negatives may explain the lower‐than‐expected correlations of the 2AFC with the MCS and 6AFC forms of the HDT in Ring and Brener (2018), as well as the fact that a higher proportion of participants were deemed interoceptive when using the MCS and 6AFC‐HDT compared to the 2AFC‐HDT.

It is noteworthy that these data speak to the proportion of participants who may be *misclassified on the basis of their delay preference*, rather than the total number of potential false negatives. A participant may be able to perceive their heartbeat and fail the 2AFC‐HDT because they have generally poor timing ability affecting their ability to make synchronicity judgments, have difficulty concentrating throughout the test, have poor motivation, are distracted by pain, made anxious by the test procedure, or any one of several other reasons. The way to identify these participants would be to employ a control task which has the same task demands as the 2AFC‐HDT but does not require the ability to perceive one's heartbeats for successful task performance. Any participant who fails both the 2AFC‐HDT and the control task would be unclassifiable; one cannot determine which of the task requirements they cannot meet, and so they cannot be classified as either interoceptive or non‐interoceptive. Unfortunately, a significant proportion of studies using the HDT (and indeed other interoceptive tasks) do not include such a control task, and therefore the percentage of participants incorrectly classified as non‐interoceptive despite being truly interoceptive is likely higher than illustrated by our study. Although we cannot say with confidence how severe the increase in those incorrectly classified will be, in a laboratory study using a non‐interoceptive control task where participants completed a version of the PAT where the heartbeats were replaced by a second tone, 14% of participants were judged to have failed the task using the updated analysis approach.

The problem with using predetermined delays in the 2AFC‐HDT does not only impact the measurement of cardiac interoceptive accuracy but also cardiac interoceptive “insight” (also called interoceptive metacognition or awareness; Suksasilp and Garfinkel 2022), a measure which has been claimed to have clinical relevance (e.g., Nord and Garfinkel 2022). Interoceptive insight refers to the degree of correspondence between an individual's assessment of their interoceptive accuracy and their actual interoceptive accuracy. When assessed with the 2AFC‐HDT, it is typically measured as the correspondence between accuracy of judgments and confidence in those judgments, across trials (e.g., Livermore et al. 2022). The use of predetermined delays affects the calculation of both accuracy on the 2AFC‐HDT and confidence judgments on each trial. For example, “the participant was directed to respond to whether the tones were in or out of time with their heartbeats, and how confident they were in that answer using a VAS scale ranging from ‘total guess’ to ‘complete confidence’ on a scale of 1 to 10” (from Livermore et al. 2022, 2292). A participant who can accurately perceive their heartbeat may perceive neither of the delays as being synchronous but still respond synchronous to whichever of the two series is more synchronous with their heartbeat, resulting in a consistent selection of, for example, the delay predetermined to be synchronous. This participant would be fully justified in responding with little confidence that the tones were synchronous, even when they are judged to be perfectly accurate. The participant would be judged as lacking interoceptive insight, even though their interoceptive insight is perfect.

Despite the utility of these data, the results are limited by gaps in our knowledge that provide critical directions for future research. First, there is little knowledge regarding the temporal acuity of heartbeat perception, which could possibly vary within and across participants. Whilst we relied on current knowledge from Brener and Kluvitse (1988; see Brener and Ring 2016) and examined results across multiple temporal windows informed by these data, future research should examine the precision with which individuals perceive heartbeat‐tone synchrony. Second, although our results provide an illustration of how the timing of heartbeat perception as measured by the PAT may impact performance on the 2AFC‐HDT, scores on the two tasks were not directly compared. Further research is therefore required to assess how individuals behave across different measures of cardiac interoceptive accuracy (e.g., MCS, 6AFC, PAT, 2AFC‐HDT). For such studies, tasks should be administered alongside appropriately matched control tasks and would ideally be administered using an ECG to circumvent potential issues relating to pulse arrival time. Relatedly, the PAT is a new task, and further work is required to explore its reliability and convergent and discriminant validity. However, as one of the most promising new tasks of cardiac interoceptive accuracy (Desmedt et al. 2023), and one of the only measures that samples the perception of heartbeat‐tone synchrony across the entire cardiac cycle, this measure was the most appropriate choice for the present study. Third, although in this study we sought to quantify the potential impact of individual differences in the timing of heartbeat perception on cardiac interoceptive accuracy, future research should explore impacts on other dimensions (e.g., interoceptive insight; cardiac timing effects; Suksasilp and Garfinkel 2022). Indeed, it remains a possibility that the lack of a correlation between ‘preconscious’ cardiac timing effects (where presentation of stimuli at systole and diastole impacts processing of those stimuli) and measures of cardiac interoceptive accuracy may be partly explained by individual differences in the timing of heartbeat perception. Finally, although not a limitation, there remains limited evidence of the stability of delay preferences over time. Although stability has been observed (see Brener and Kluvitse 1988) this has not been examined only in individuals judged to be interoceptive at both time points. Alongside work on the temporal acuity of heartbeats and pulse arrival time, such studies may be useful for assessing the degree to which delay preferences remain stable and can be used to present stimuli for circumstances where a simplified design (e.g., 2AFC using personalized delays from other measures) is essential (for a discussion of similar previous approaches using ‘personalized delays’, see Brener and Ring 2016).

To conclude, this study demonstrates that the 2AFC‐HDT may result in false negatives, whereby interoceptive participants are incorrectly misclassified as being non‐interoceptive due to individual differences in the point in the cardiac cycle at which participants perceive heartbeats. Additionally, the use of the 2AFC‐HDT is also likely to result in incorrect calculation of interoceptive insight or awareness. This is important as it affects the validity of conclusions drawn when comparing interoceptive and non‐interoceptive groups, as determined by the 2AFC‐HDT. Ideally measures should also present responses across the entire cardiac cycle, not be influenced by knowledge regarding heart rate, include control measures and account for both phase‐ and delay‐based response patterns. At present, to our knowledge, the PAT with its updated analysis approach is the only method of assessing cardiac interoceptive accuracy that meets these criteria.

## Author Contributions

**Kiera Louise Adams:** conceptualization, methodology, validation, formal analysis, writing (original draft), visualization. **Ren Palmer:** methodology, software, formal analysis, data curation, visualization. **Jonathan M. Bird:** methodology, software, data curation, writing (review and editing). **David Plans:** methodology, software, resources, data curation. **Adam Cunningham:** methodology, software, resources, data curation. **Davide Morelli:** methodology, software, resources, data curation. **Tom Piercy:** methodology, software, resources, data curation, writing (review and editing). **Ria Spooner:** investigation. **Rhea Clemente:** investigation. **Nerea Irigoras Izagirre:** investigation. **Elisa Fernandez Fueyo:** investigation. **Dawn Watling:** investigation. **Rebecca Brewer:** investigation. **Gemma Budworth:** investigation. **Dorina Cocirla:** investigation. **Mateo Leganes‐Fonteneau:** investigation, writing (review and editing). **Jennifer Todd:** investigation. **Jane E. Aspell:** investigation. **Geoffrey Bird:** conceptualization, methodology, writing (review and editing). **Jennifer Murphy:** conceptualization, methodology, investigation, writing (review and editing), supervision, project administration.

## Funding

This work was supported by K.L.A. and funded through the University of Oxford Medical Sciences Graduate School Studentship (Clarendon Fund in partnership with the University College Award and the Experimental Psychology Studentship). J.M.B. is supported by the NIHR University College London Hospitals Biomedical Research Centre. R.S. is funded by an ESRC South East Network for Social Sciences (SeNSS) PhD studentship ES/P00072X/1. R.B. is supported by the Medical Research Council, UKRI (MR/S003509/1). E.F.F. was funded by Royal Holloway, University of London, and the LIDo/BBSRC Studentship (BB/T008709/1). M.L.F. was funded through the Center of Alcohol and Substance Use Studies pilot grant at Rutgers University and the National Fund for Scientific Research of Belgium. J.T. and J.E.A. were funded through the Pain Challenge Research Award from Versus Arthritis (ref 22461).

## Conflicts of Interest

G.B. and D.P. have a financial interest in Huma Therapeutics. J.M. and G.B. have completed paid work on interoception for Healios.

## Supporting information


**Supporting Information: S1** Inclusion and exclusion criteria for each study.
**Supporting Information: S2:** Demographic information for each study before only interoceptive participants were selected.
**Supporting Information: S3:** Full instructions for the PAT.
**Supporting Information: S4:** Descriptive statistics of the different IBIs (secs), taken across all trials for all interoceptive participants.
**Supporting Information: S5:** Delay choices for each participant.
**Supporting Information: S6:** The number of participants who passed the screener who selected delays aligned with the predefined synchronous and asynchronous delays used in the 2AFC‐HDT.

## Data Availability

The data that support the findings of this study will be uploaded to OSF upon acceptance of the article.

## Endnotes

^1^
Note that we use cardiac interoceptive accuracy to refer to measures that examine the correspondence between subjective perception and objective physiological indices for consistency with the wider field, but acknowledge that this terminology remains contested (Desmedt and Van den Bergh 2024).

^2^
Note that we use the term “interoceptive” as a shorthand to refer to individuals who exhibit above chance performance on a particular task at a particular point in time.

^3^
Although the 150 ms window technically allows for overlap between the synchronous and asynchronous options at fast heart rates, this did not occur in practice.
