# Supplementary material for: Individual Differences in Heartbeat‐Tone Synchronicity Judgments Suggest the Two‐Alternative Heartbeat Detection Task Is a Poor Test of Cardiac Interoceptive Accuracy and Insight
Source: Psychophysiology. 2026 May 13;63:e70310. doi: 10.1111/psyp.70310 (PMC13170068; doi:10.1111/psyp.70310)
Supplement: Supplementary file 1 — Supporting Information: S1 Inclusion and exclusion criteria for each study. Supporting Information: S2: Demographic information for each study before only interoceptive participants were selected. Supporting Information: S3: Full instructions for the PAT. Supporting Information: S4: Descriptive statistics of the different IBIs (secs), taken across all trials for all interoceptive participants. Supporting Information: S5: Delay choices for each participant. Supporting Information: S6: The number of participants who passed the screener who selected delays aligned with the predefined synchronous and asynchronous delays used in the 2AFC‐HDT. [file PSYP-63-e70310-s001.docx]

**Supplementary Materials**

*Supplement 1:*

*Inclusion and exclusion criteria for each study*

| Study | Inclusion criteria | Exclusion criteria | Filtering criteria | Ethics approval | Recruitment methods |
| --- | --- | --- | --- | --- | --- |
| *Study 1 (Todd et al., 2024)* | 29 to 65 years old  Owning a compatible smartphone*  Willingness to download the free application | Significant hearing loss that prevents response to auditory stimuli  Presence of a diagnosed cardiac condition  Pregnancy  Previous diagnosis of a pain condition  Presence of chronic pain symptoms in the last three months | Removal of participants with duplicate IP addresses  And who completed the study in a time that was implausible (i.e., <10 minutes) or did not provide sufficient data (i.e., 80% complete). | Anglia Ruskin University (PSY-S19-023) | Prolific Academic |
| *Study 2 (from unpublished study examining interoception and empathy, dataset managed by jennifer.murphy@surrey.ac.uk)* | Enforced by experimenter in laboratory: 18 years old or older | Uncorrected visual or auditory issues  Cardiac problems  Mental health conditions |  | Royal Holloway University 3090 | Posters around Royal Holloway University Campus |
| *Study 3 (from unpublished study examining attention to interoceptive signals, dataset managed by jennifer.murphy@surrey.ac.uk* | Enforced by experimenter in laboratory: 18 years old or older | Uncorrected visual or auditory issues  Cardiac problems  Mental health conditions |  | Royal Holloway University 2186 | Posters around Royal Holloway University Campus |
| *Study 4 (dataset managed by Mateo Leganes-Fonteneau)* | Aged 18–35 years; at least one night of heavy drinking in the past 30 days (4 or 5 alcoholic drinks in less than 2 h for females and males, respectively); consume more than 5 drinks per week; one hangover in past 30 days; one blackout episode in the past 6 months. Access to an Apple iPhone meeting eligibility requirements | No self- reported history of cardiovascular or mental health disorder. |  | Rutgers University Institutional Review Board Pro2021002431 | Word of mouth, online fora (i.e., reddit), student lists, and through flyers pasted in bars and breweries in Continental USA. |
| *Other studies (dataset managed by jennifer.murphy@surrey.ac.uk)* | Enforced in Testable: aged 18 to 60 years old, English as a first language, passed screener  General: owns a compatible smartphone or iPad  Cisgender | Mental health conditions  Cardiac conditions |  | Royal Holloway University 3090 and 3056 | Testable |
|  |  |  |  |  |  |

*Note.**Compatible smartphones include any Apple iPhone with one camera lens running iOS 13 or higher. For Study 4, we only included data from the initial baseline session where participants were told to refrain from drinking alcohol for 24 h. Testable and Prolific are both are online platforms for participant recruitment.

*Supplement 2:*

*Demographic information for each study before only interoceptive participants were selected*

| Study | Sex | Age in years (average / S.D.) | Presence of mental health condition | Presence of physical health condition | Missing data (N) | Interoceptive at BF30 (N / %) |
| --- | --- | --- | --- | --- | --- | --- |
| *Study 1 (Todd et al., 2024*  *Pain free sample)*  *N= 84* | Male: 0.0%  Female: 100.0%  Other: 0.0% * | 40.1 (8.3) | 13.3% | 28.9% | 1 | 19 (22.6%) |
| *Study 2*  *N= 57* | Male: 28.1%  Female: 71.9% | 27.2 (12.9) | 0% | Data not collected | 0 | 14 (24.6%) |
| *Study 3*  *N= 91* | Male: 38.5%  Female: 61.5% | 23.3 (7.6) | 19.8% | 13.2% | 0 | 23 (25.3%) |
| *Study 4*  *N= 10* | Male: 40%  Female: 60% | 23.5 (3.3) | Data not collected | Data not collected | 0 | 3 (30.0%) |
| *Other studies*  *N= 115* | Male: 46.9%  Female: 53.1% | 30.9 (9.4) | 0.9% (data not collected for 10.4%) | Data not collected | 2 | 27 (23.5%) |

*Note:* This table reflects the demographics of the initial samples, before only those participants who were interoceptive were selected. For the demographics of the interoceptive sample, see Table 3 in the main manuscript

* These studies collected information on gender rather than sex

*Supplement 3:*

*Full instructions for the PAT*

Screen 1: “How well can you match a sound with your heartbeat? Let’s find out!”

Screen 2: “First, find a quiet place where you can sit comfortably upright with your earphones on for around 10 minutes.”

Screen 3: “You will be asked to place your finger on the phone camera (on the back) so that the app can read your heartbeat. Once your finger is in position, you will hear a series of sounds. Each sound actually represents one of your own heartbeats!”

Screen 4: “It might seem like there is a delay between the sounds and the heartbeats you feel. Play the video below to hear an example! [VIDEO 1]”

Screen 5: “In order to rectify the delay, you will be asked to move a dial until the sounds are in sync with your heartbeats. Play the video below to hear an example! [VIDEO 2]”

Screen 6: “If you move the dial to the right, the delay between the heartbeat and the sound will get longer; if you move it to the left, the delay will get shorter.”

Screen 7: “Want to know how this might look like? Press “continue” to watch a short tutorial.”

Screen 8: [VIDEO 3]

Screen 9: “After you have matched the sound with your heartbeat, you will be asked how sure you are about the answer you gave. Press “confirm” then “continue” to start the following trial. In this task, there will be 20 trials in total. [EXAMPLE OF THE CONFIDENCE SCALE]”

Screen 10: “You can feel your heartbeat in different places in your body, such as your chest or yours fingers. You will be asked to indicate where you felt your heartbeat on a body map (like the one below) once every 5 trials. You can choose any of the highlighted body parts or you can select “nowhere” if you haven’t felt your heartbeat in any particular place. [EXAMPLE OF THE BODY MAP]”

Screen 11: “For the duration of this task, please do not actively try to feel your pulse with your hand; we are only interested in what you feel! When you are ready to start, please sit comfortably upright with your earphones on and press “continue”.”

Screen 12: [HEART RATE BASELINE READING]

Screen 13: “You will now get a chance to do two practice trials. Focus on feeling your heartbeat and try to match the sounds to your own heartbeat.”

*Supplement 4*

*Descriptive statistics of the different IBIs (secs), taken across all trials for all interoceptive participants*

|  | Mean | SD | Median | Min | Max |
| --- | --- | --- | --- | --- | --- |
| Final IBI | 0.821 | 0.102 | 0.819 | 0.605 | 1.070 |
| Median IBI final angle array | 0.830 | 0.119 | 0.836 | 0.593 | 1.212 |

*Note.* The values here reflect the statistics for comparing the values across all trials for all participants. The average difference between the final angle array average IBI and the final IBI was -0.002s (SD= 0.019s), max difference -0.136s. Thirty (4.1%) trials had a difference larger than 50ms. The correlation across all trials was large (*r*(731) = .99, *p* <.001).

Supplement 5

*Delay choices for each participant*

*
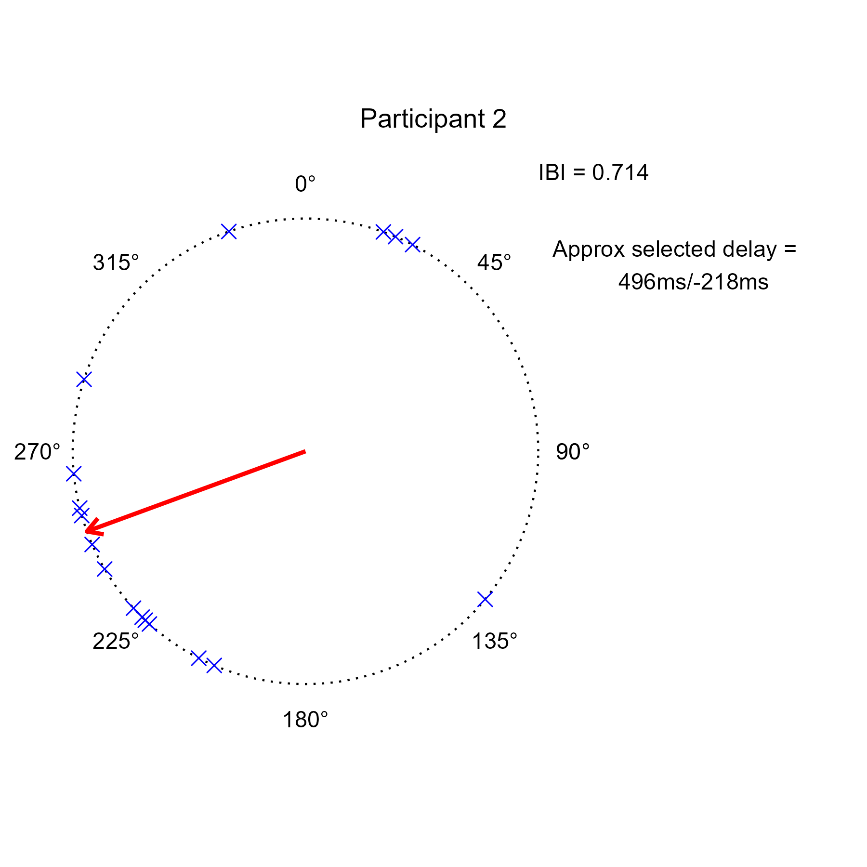

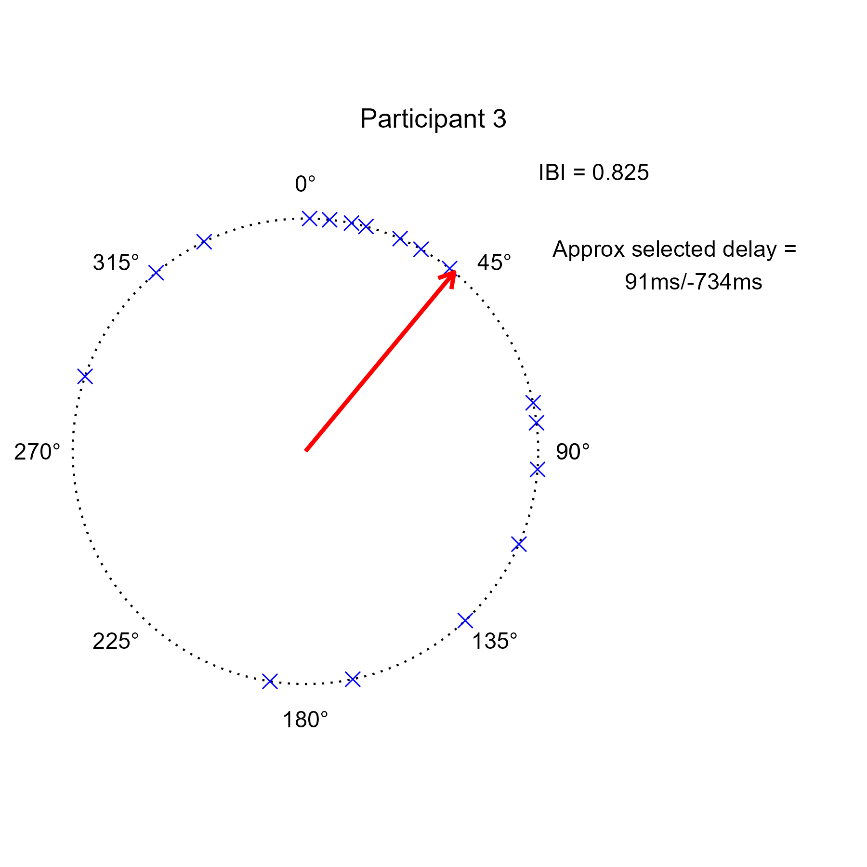

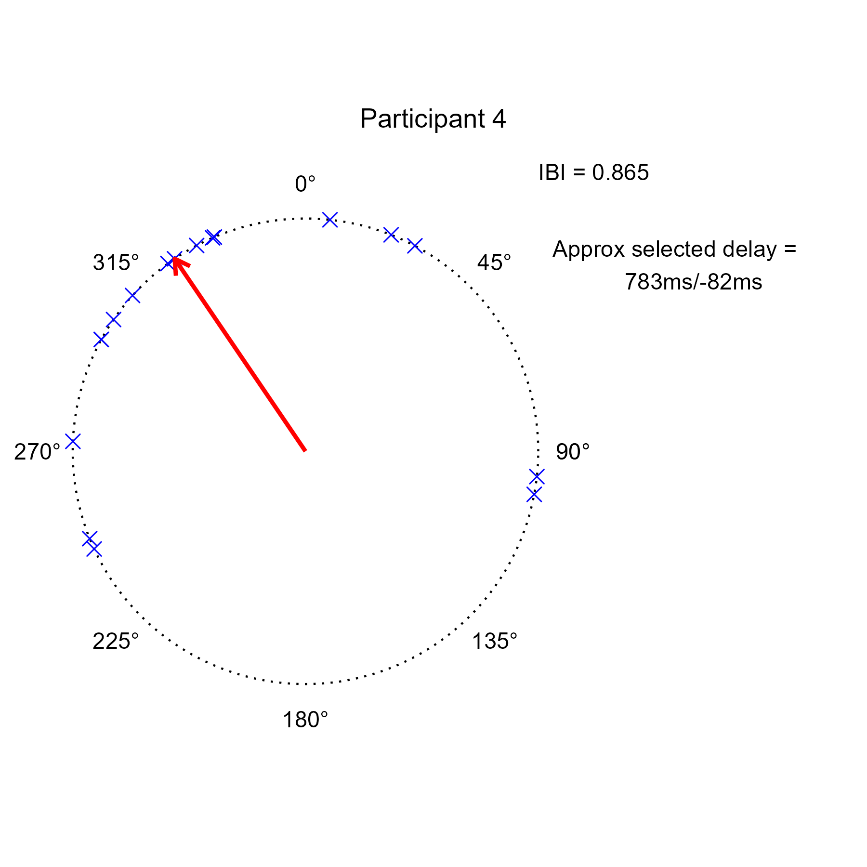

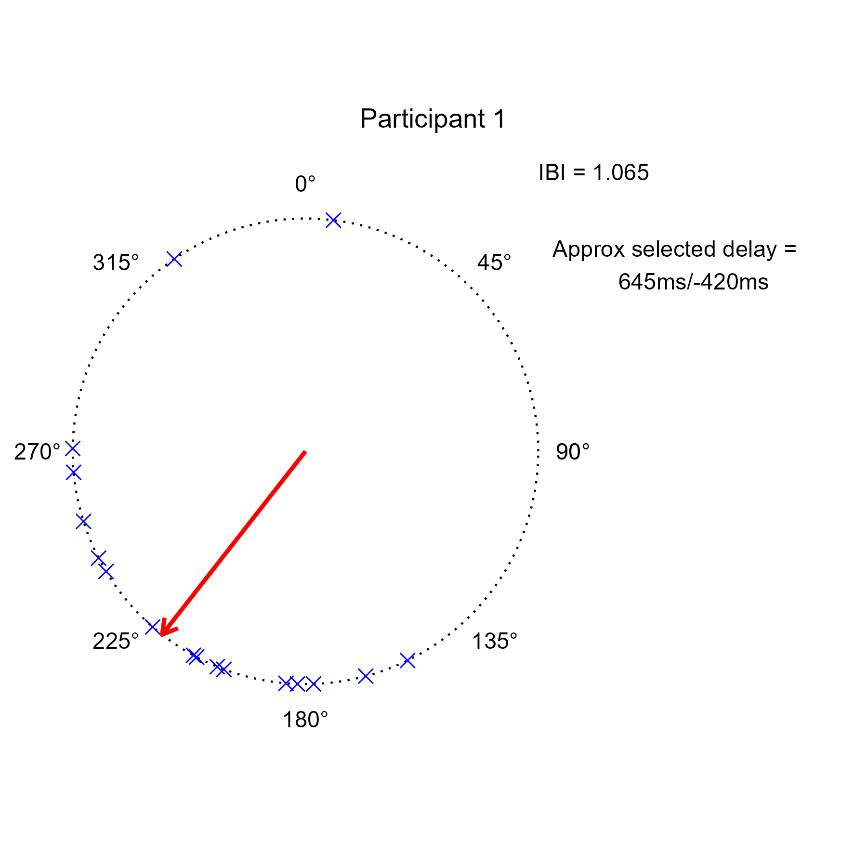

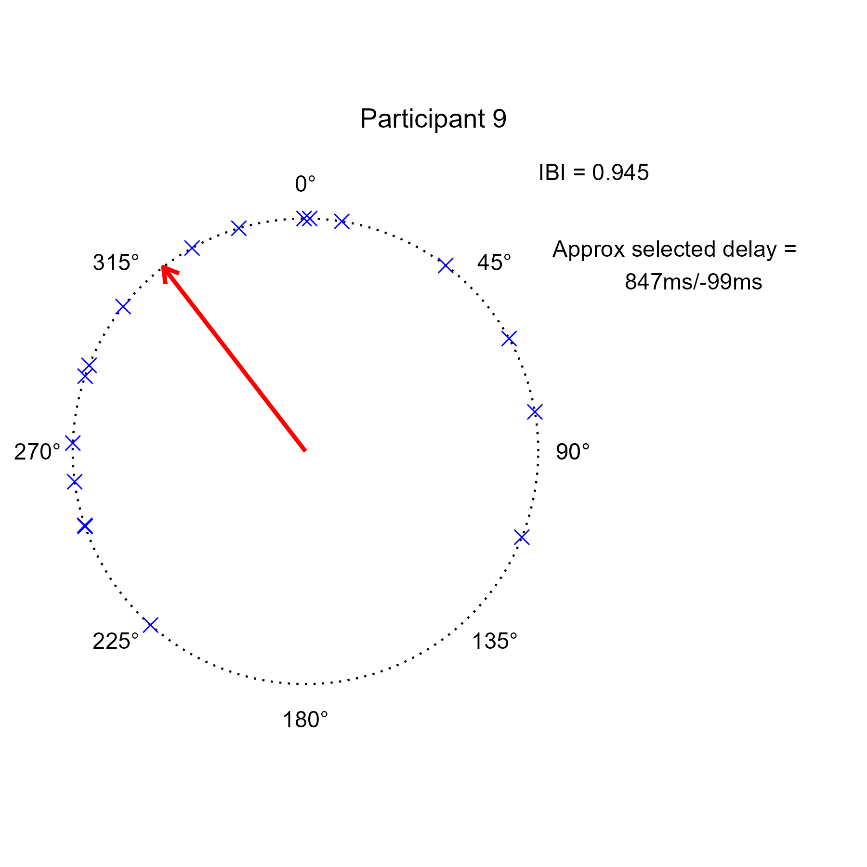

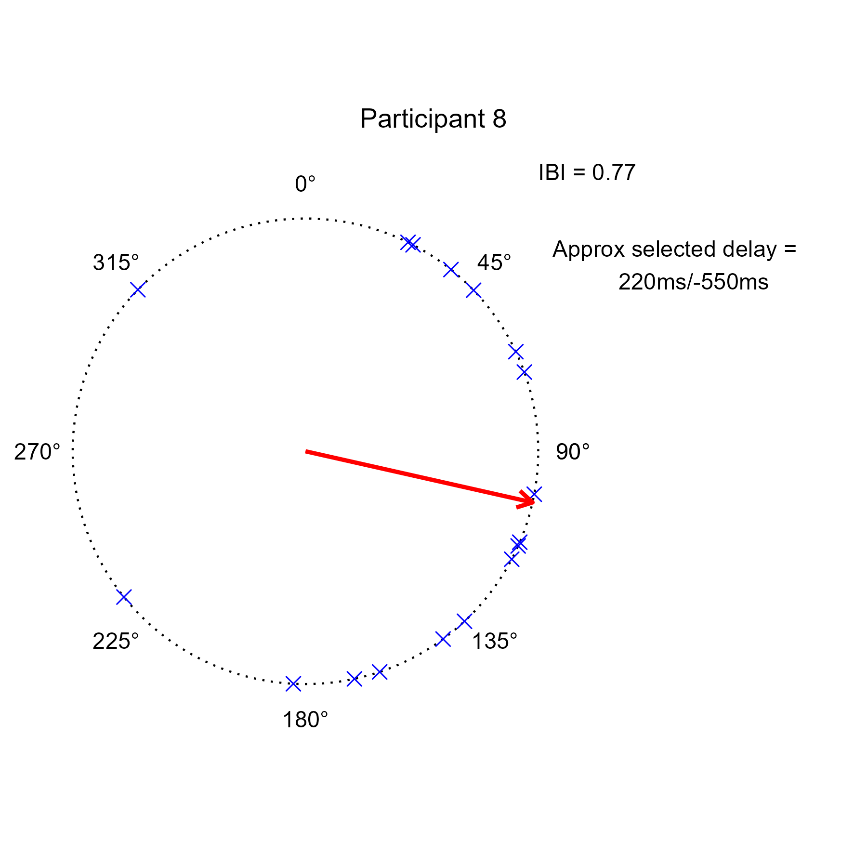

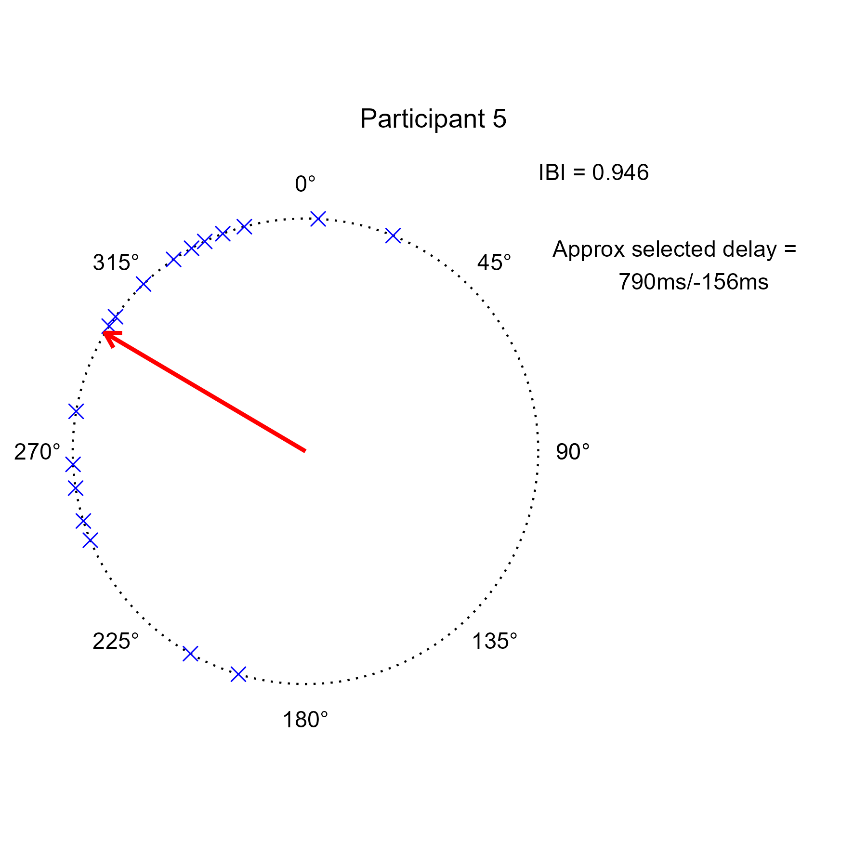

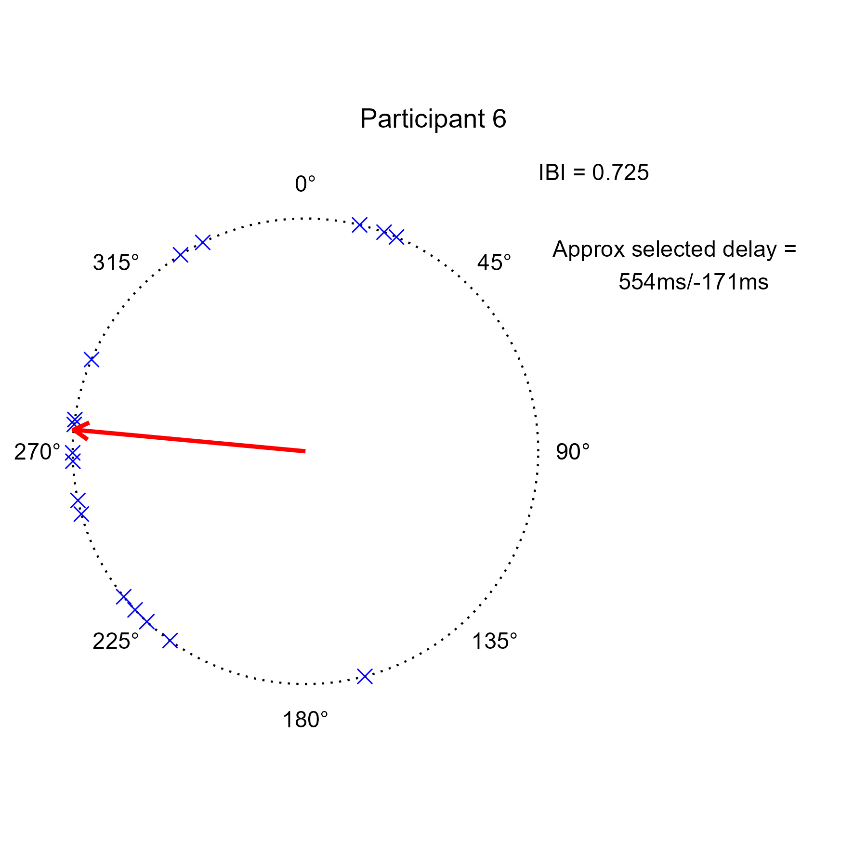

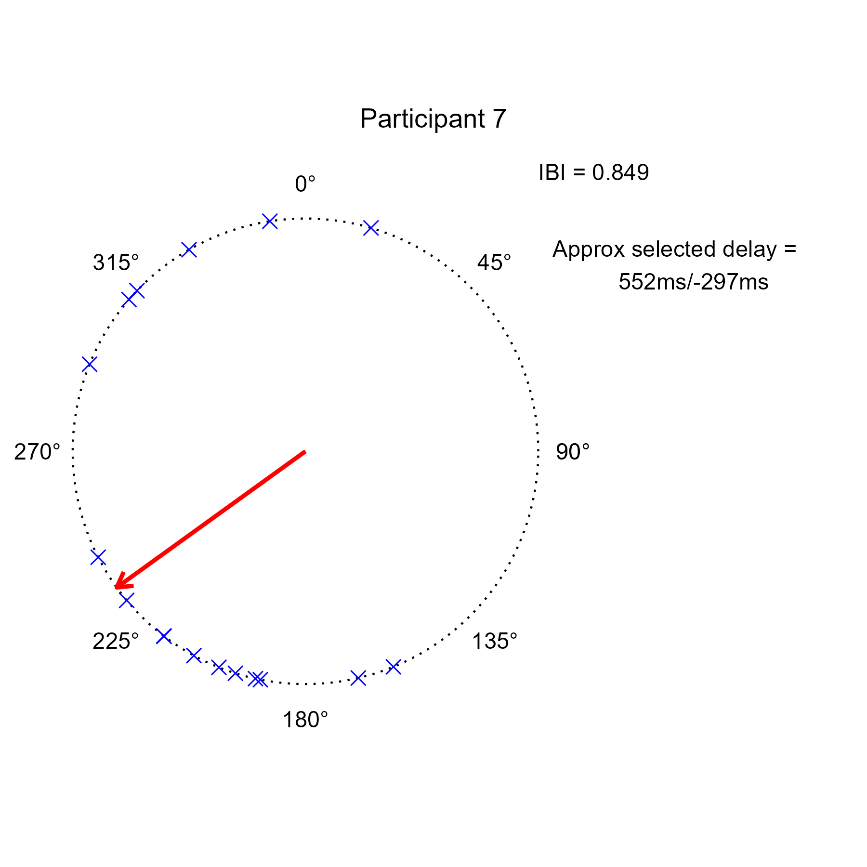

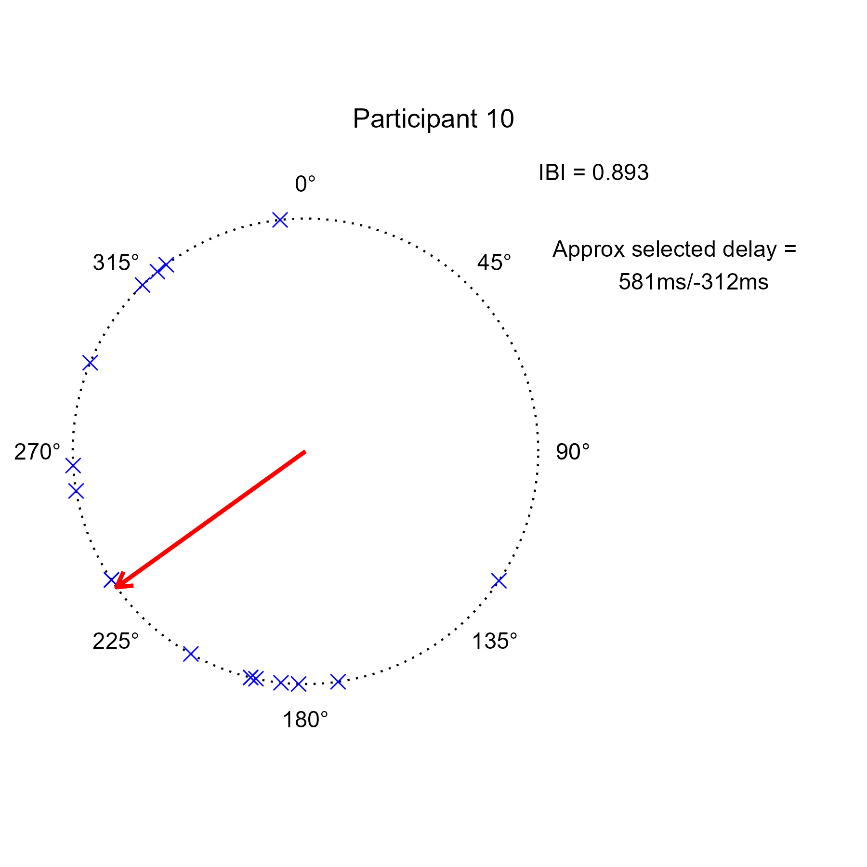

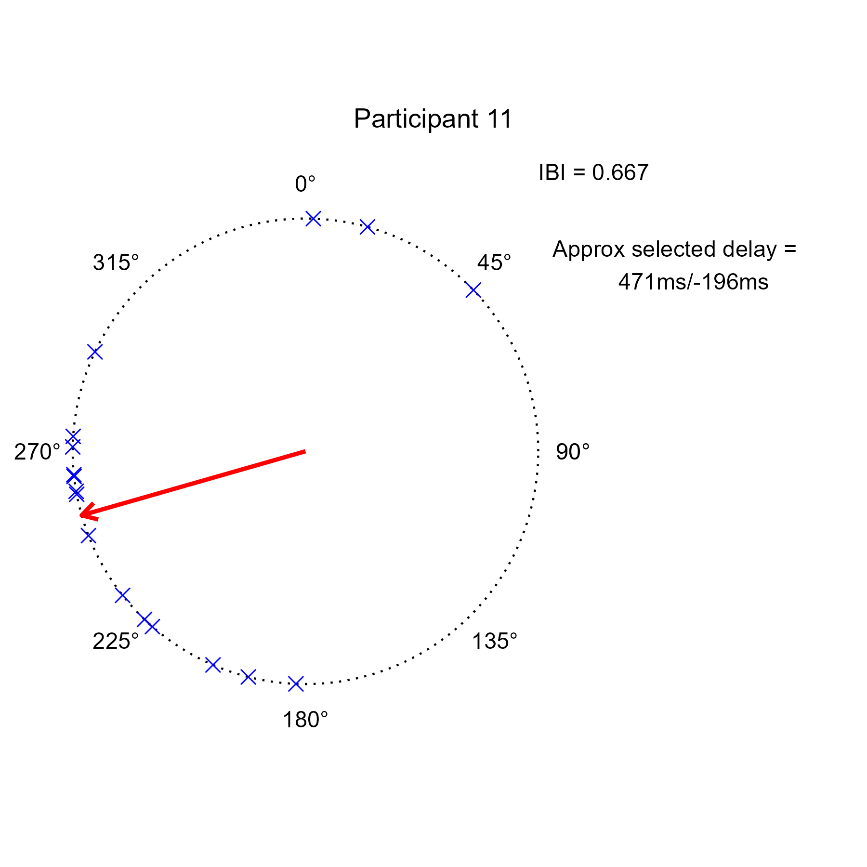

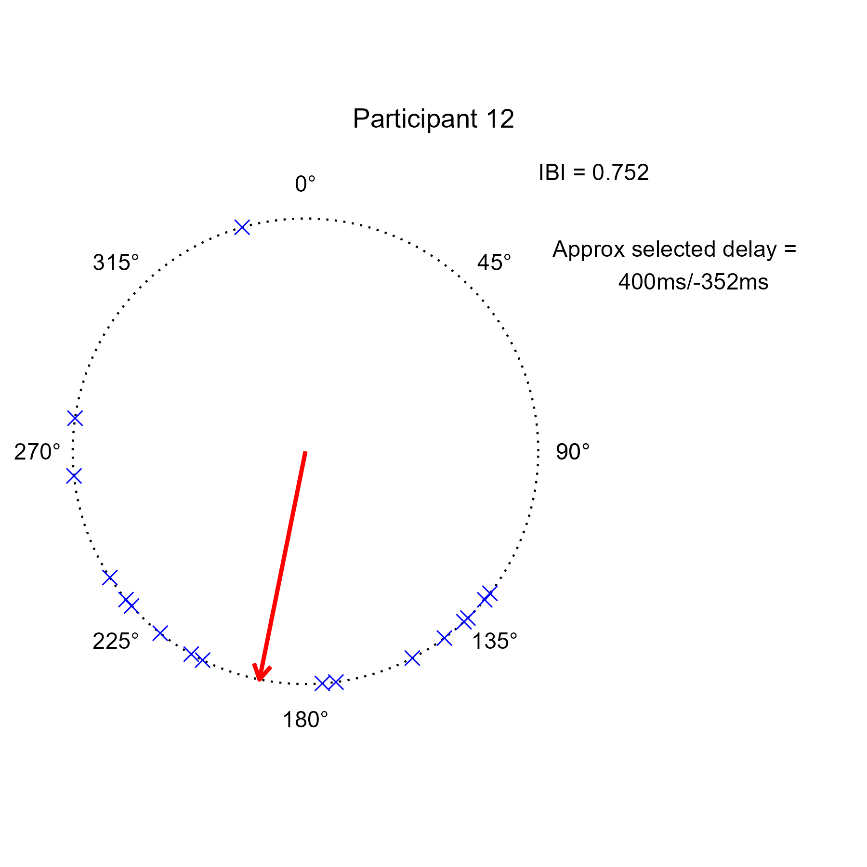

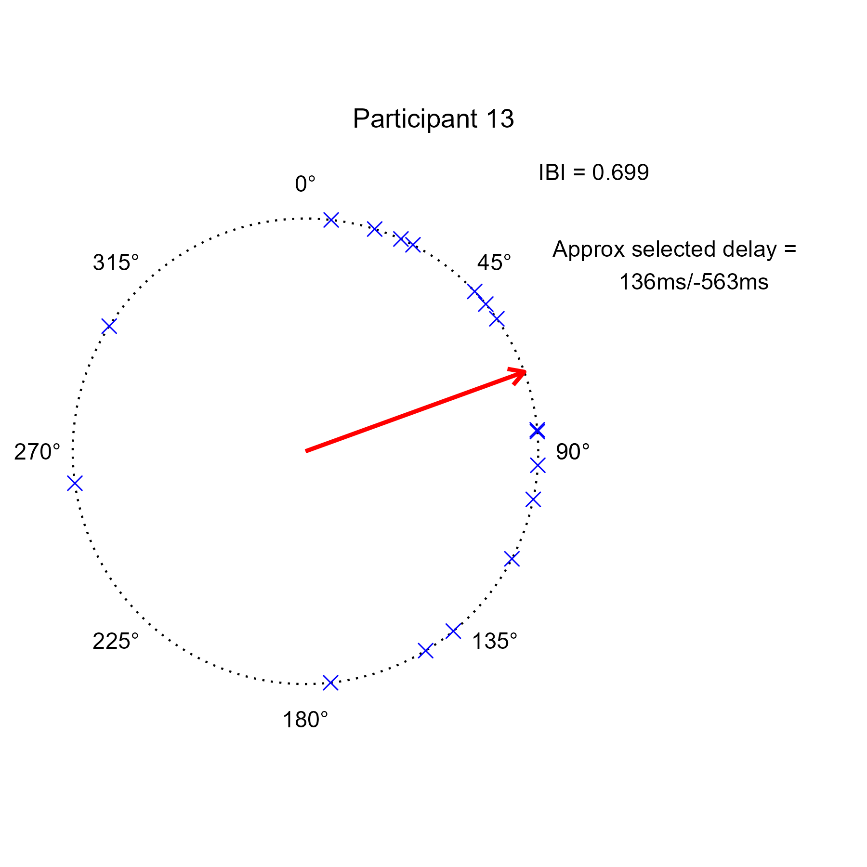

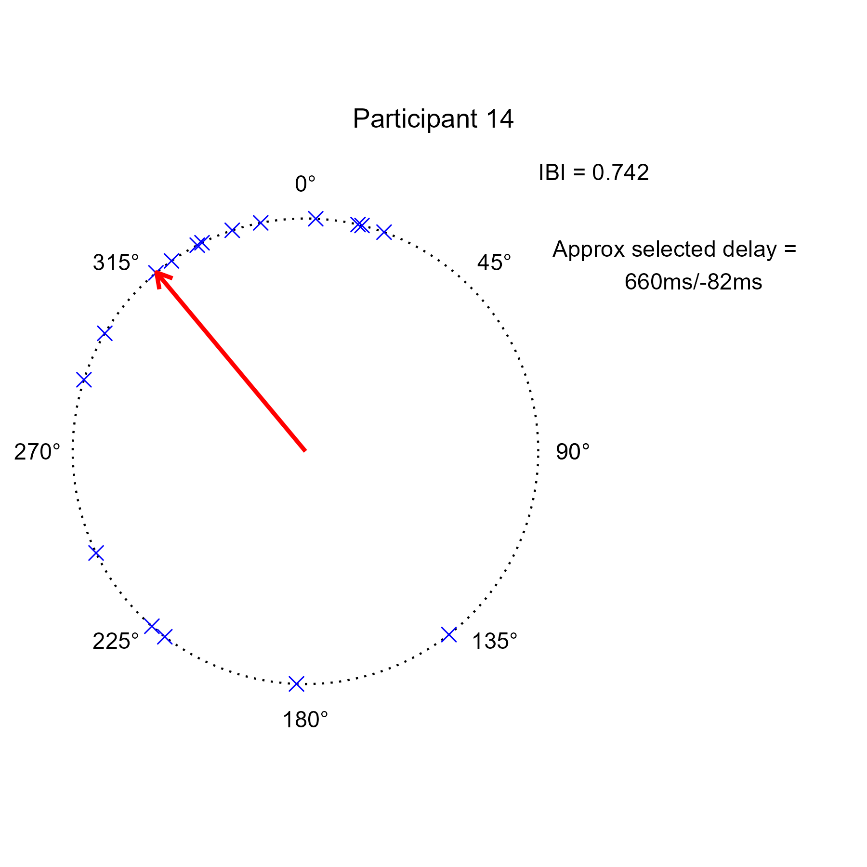

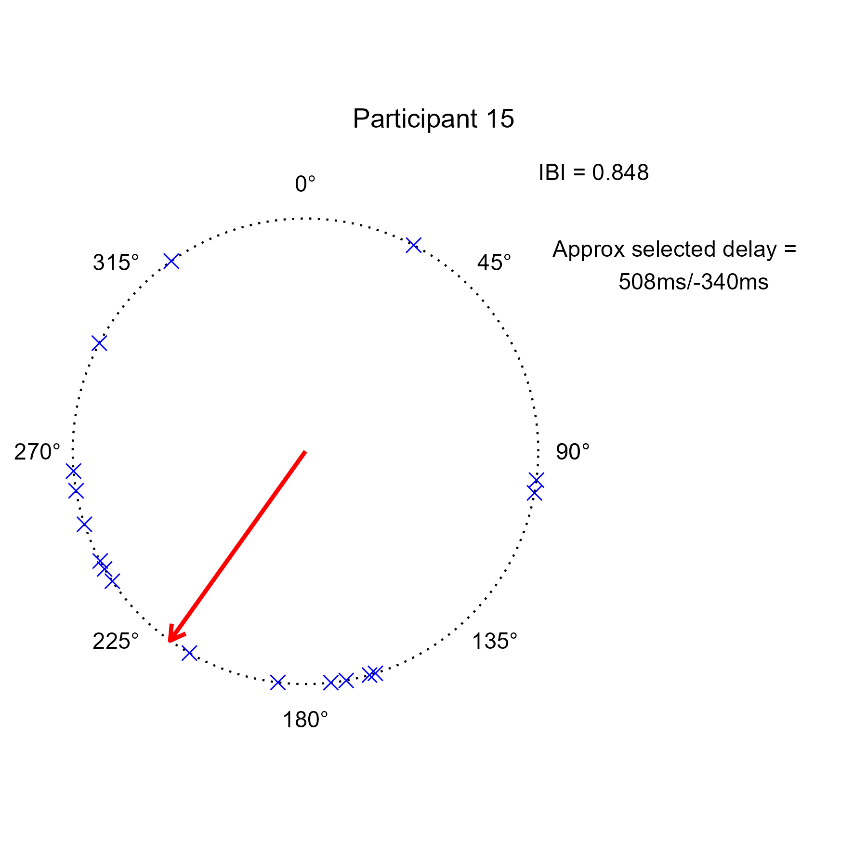

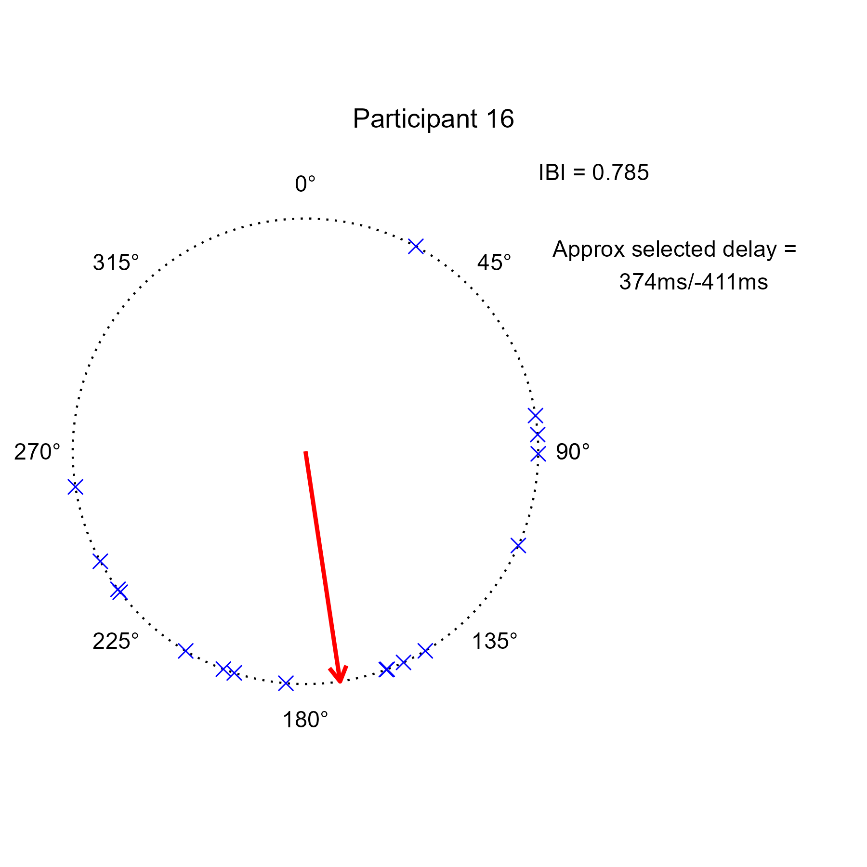

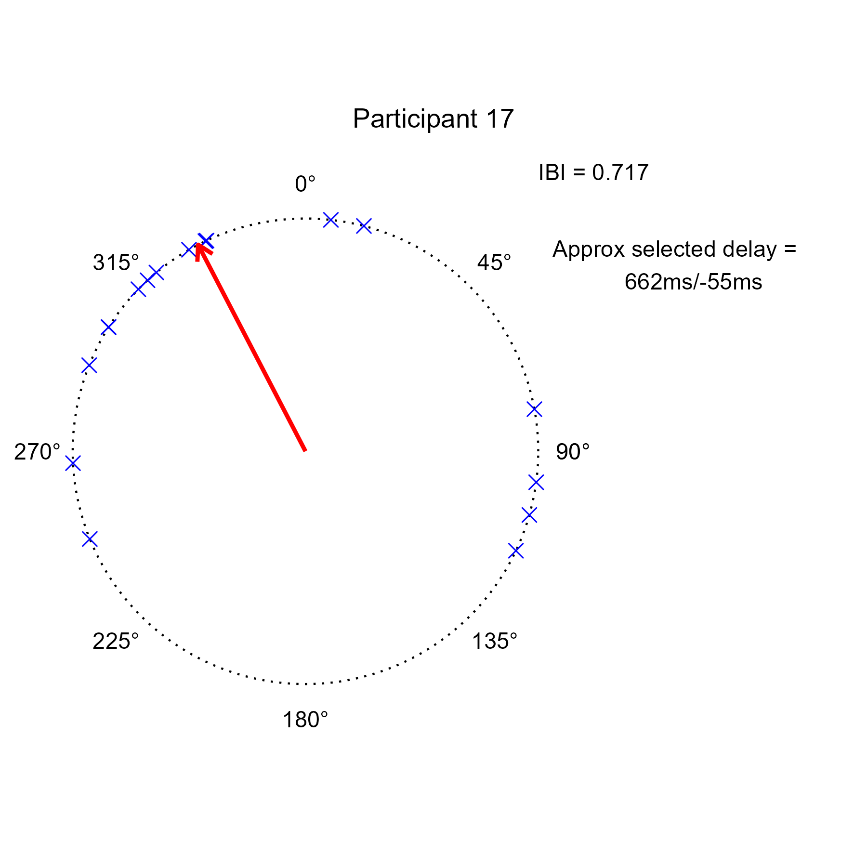

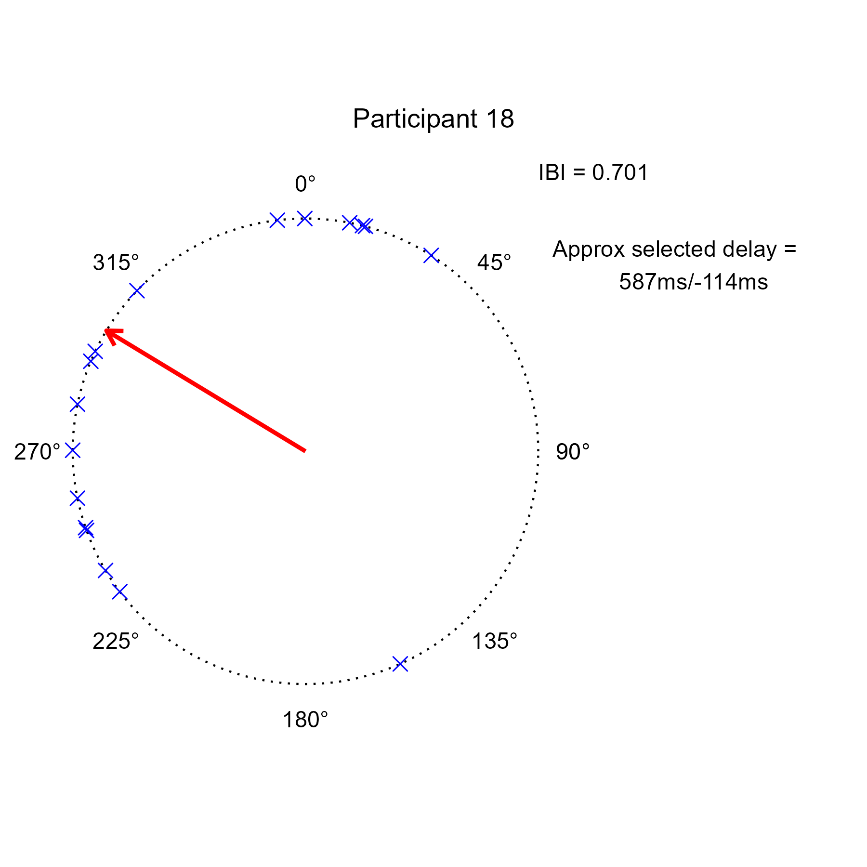

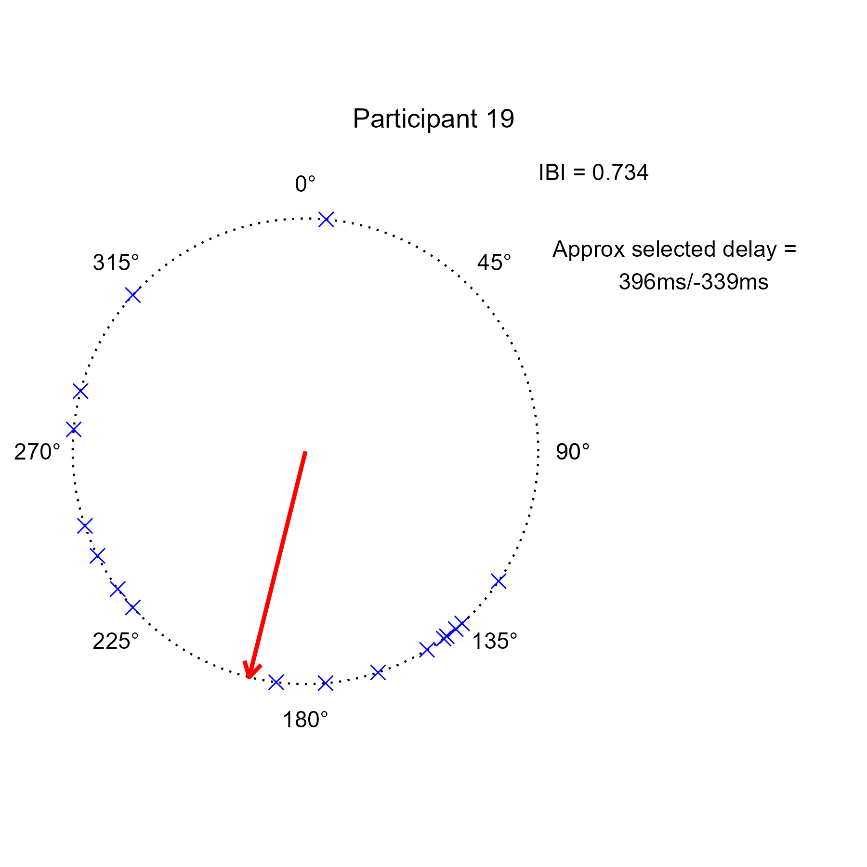

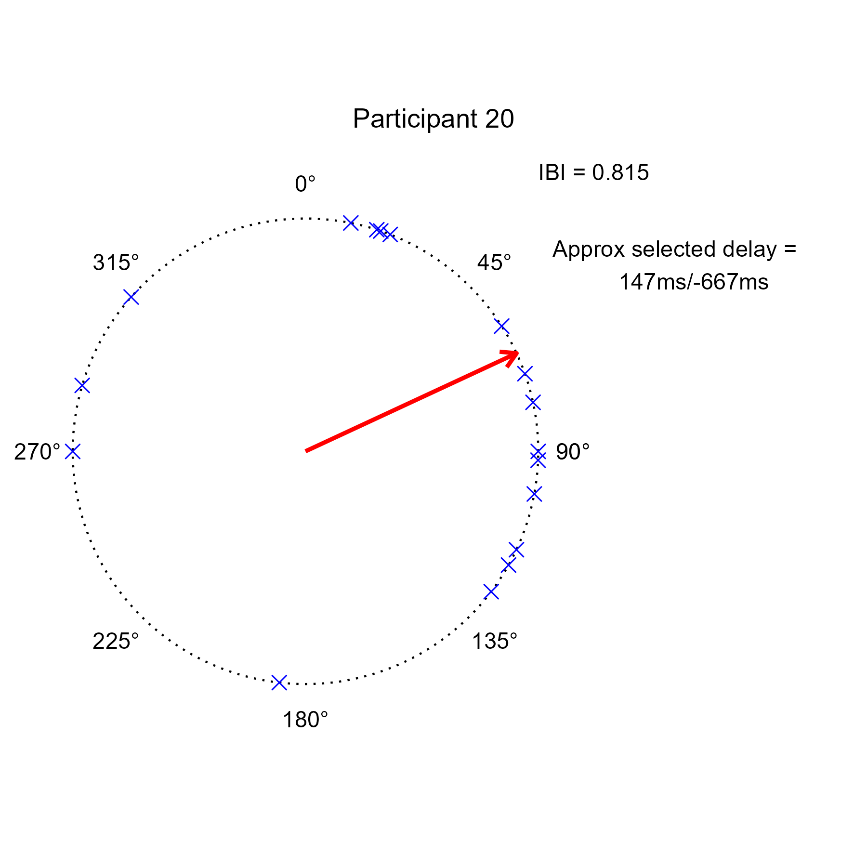

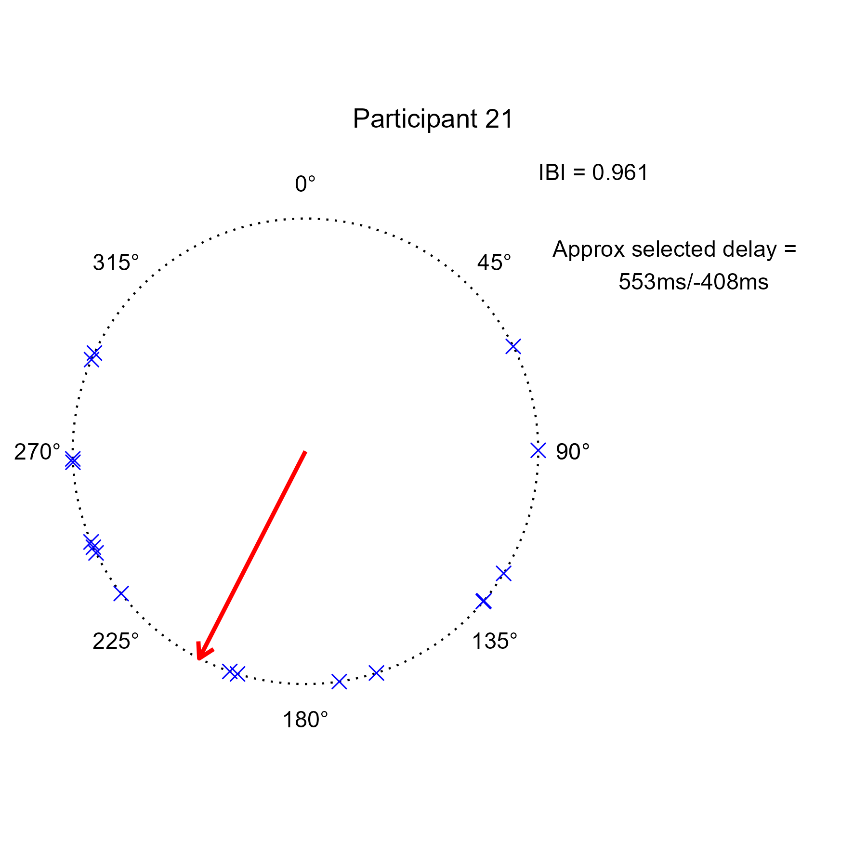

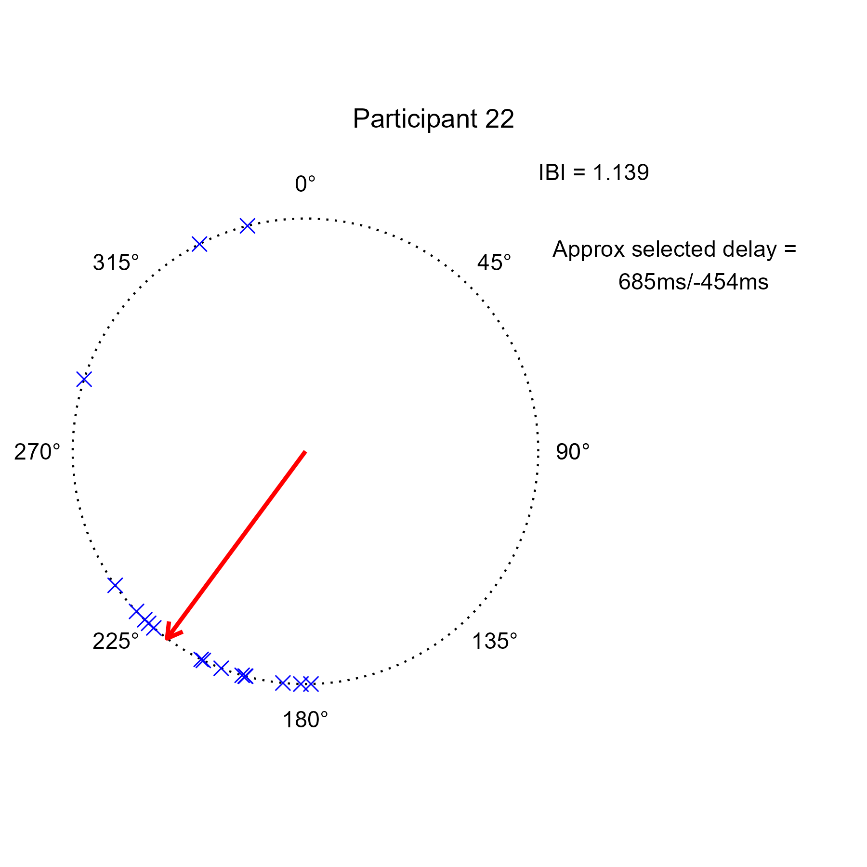

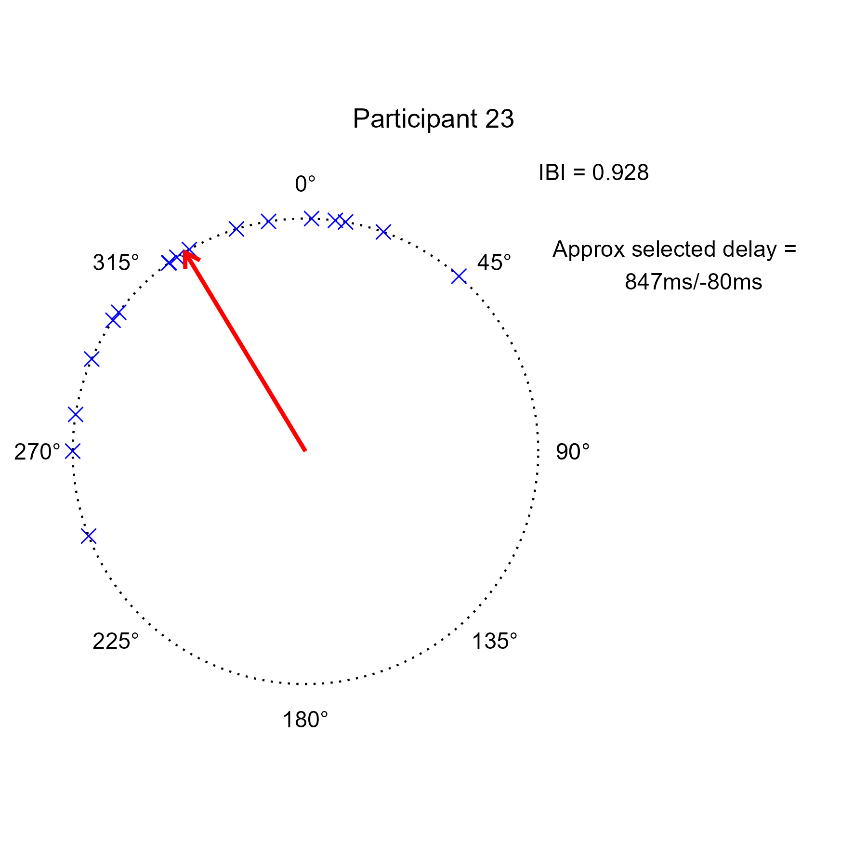

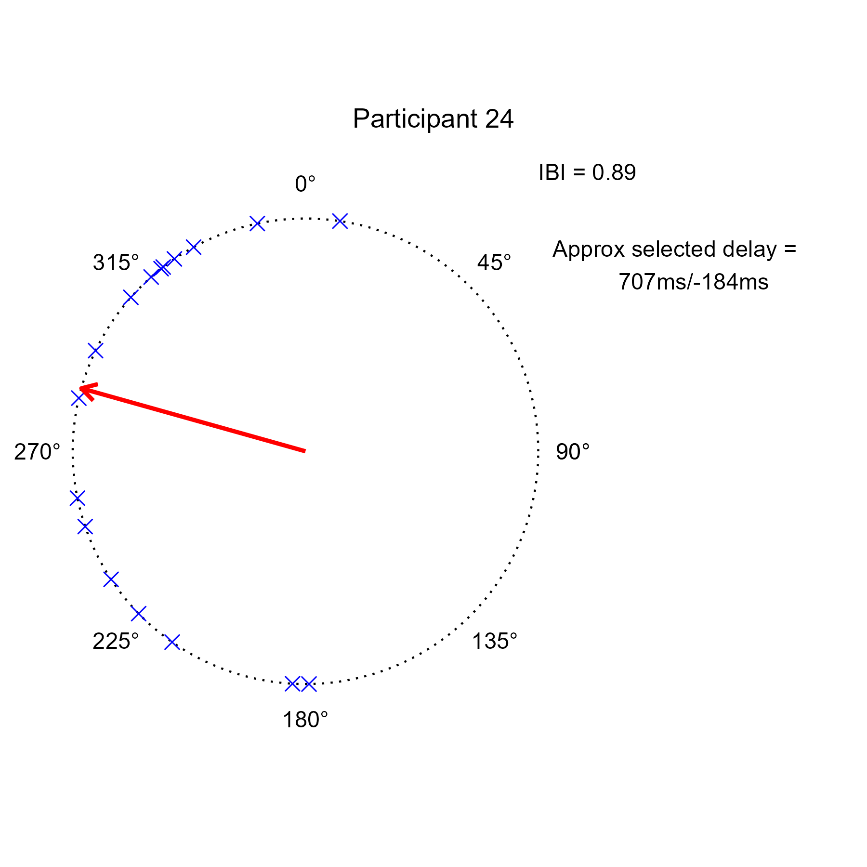

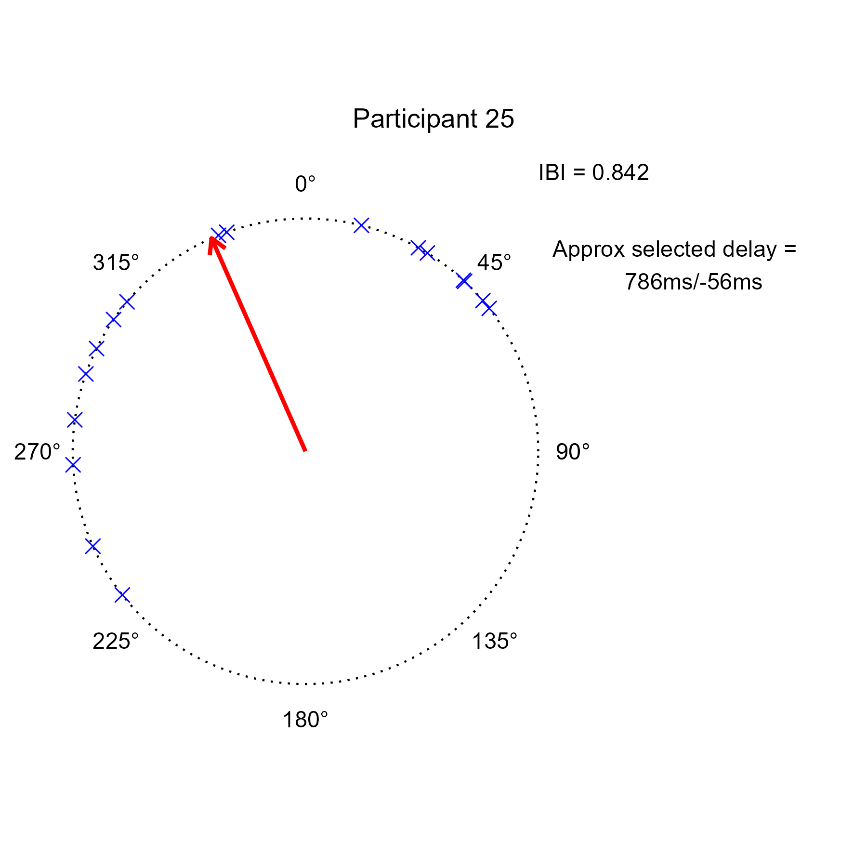

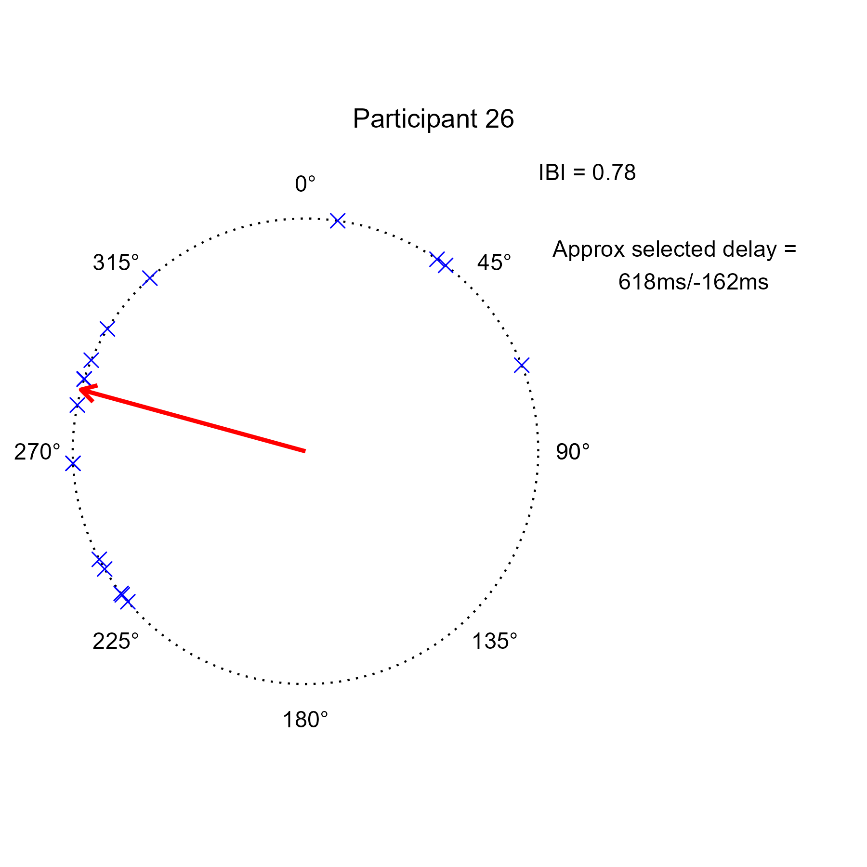

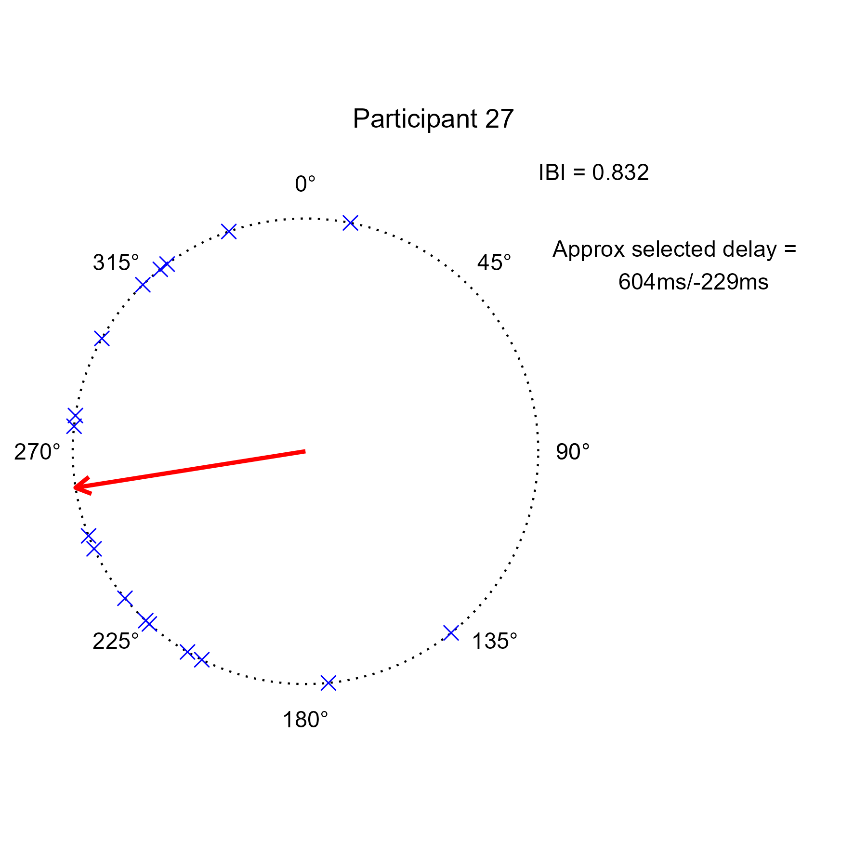

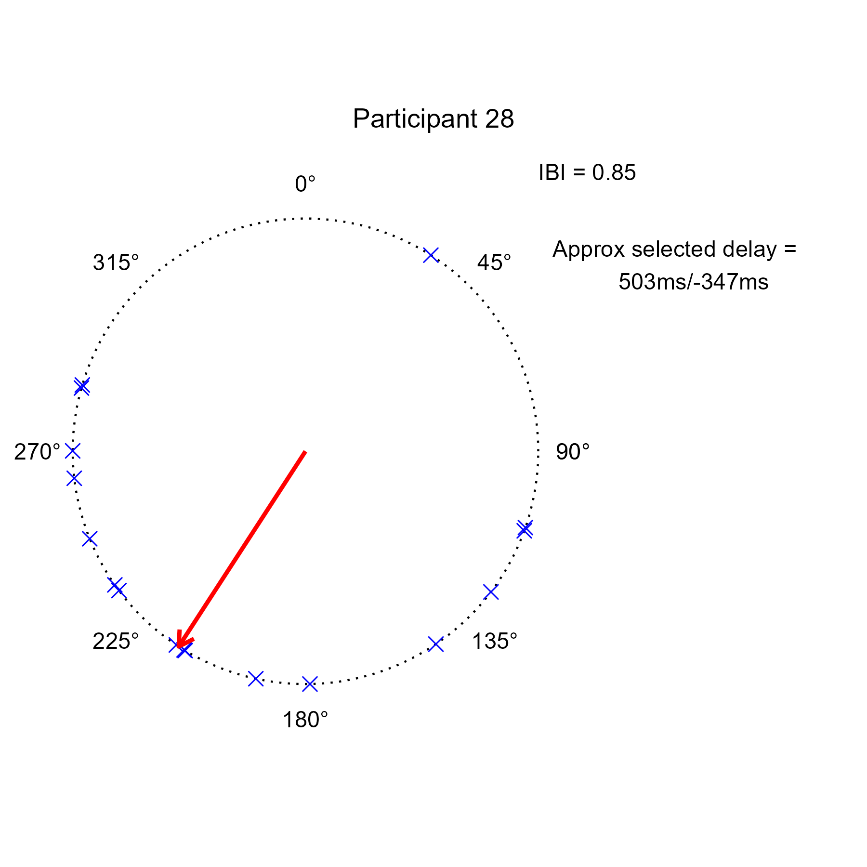

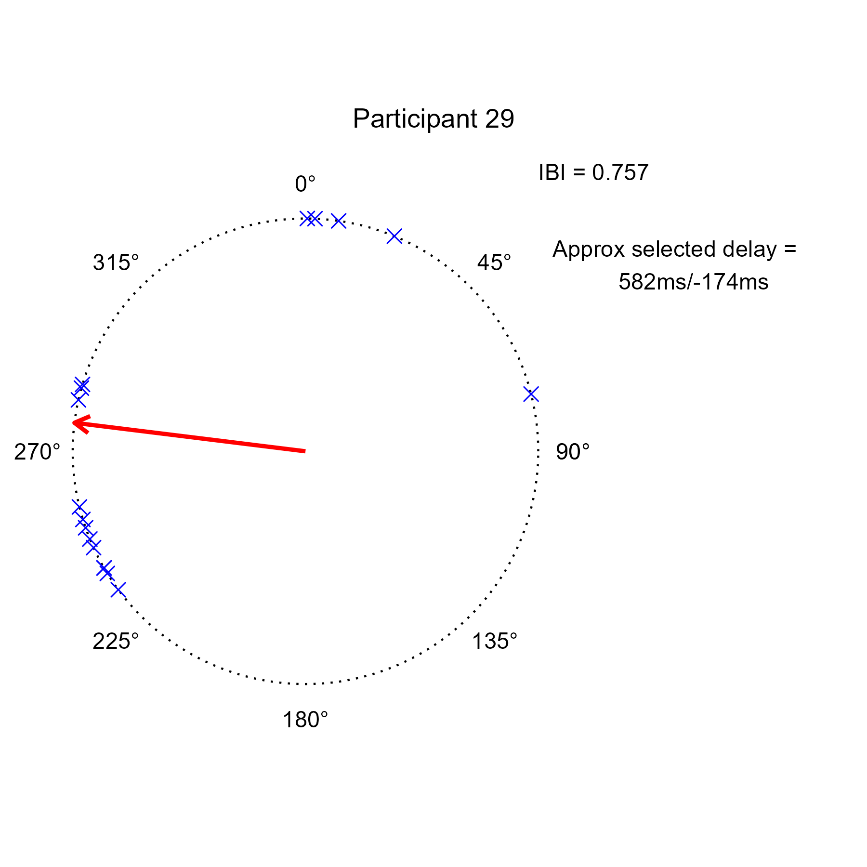

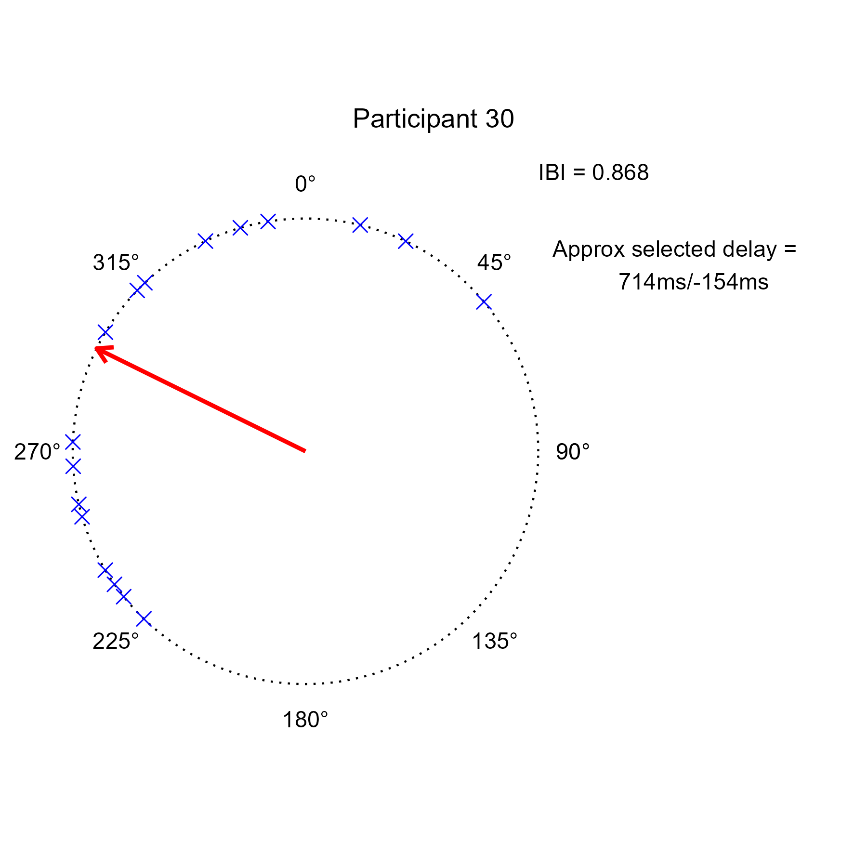

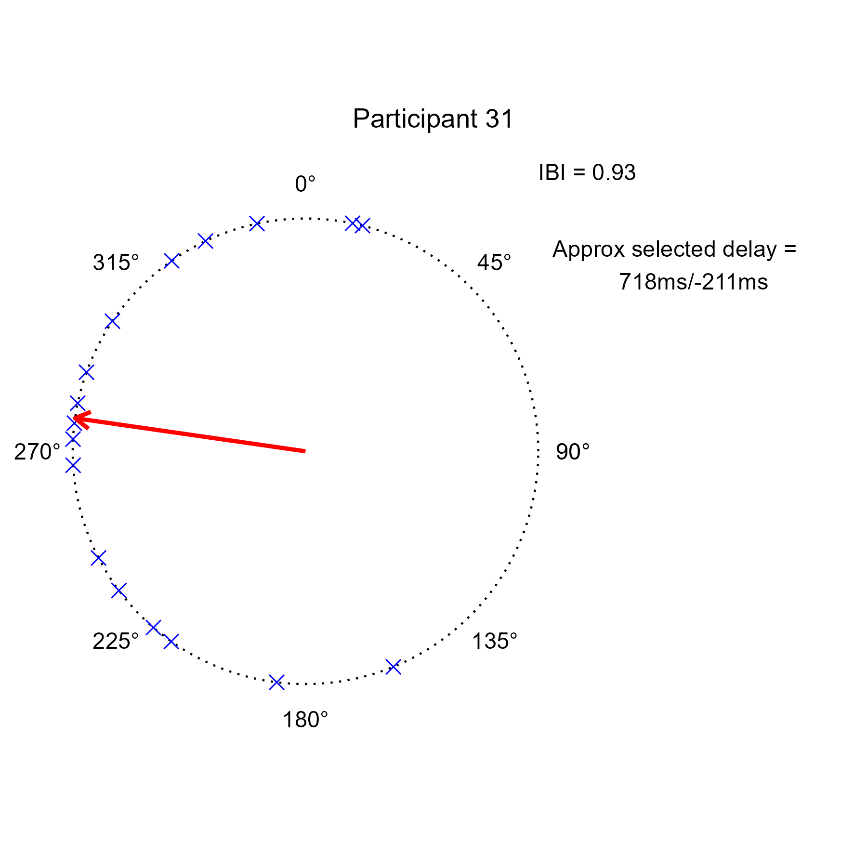

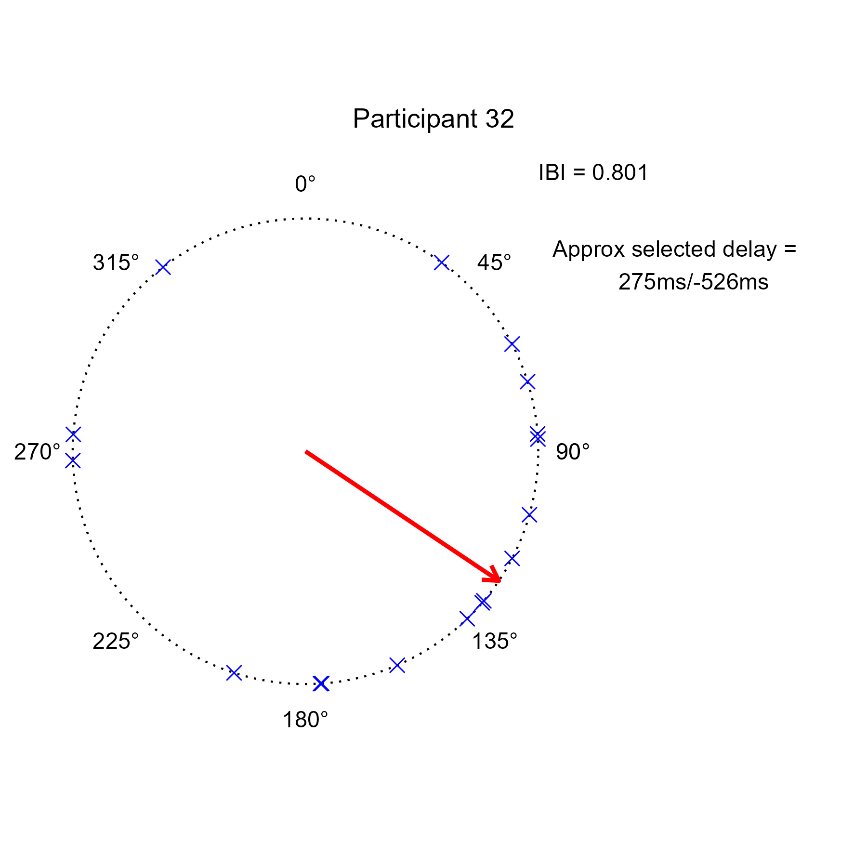

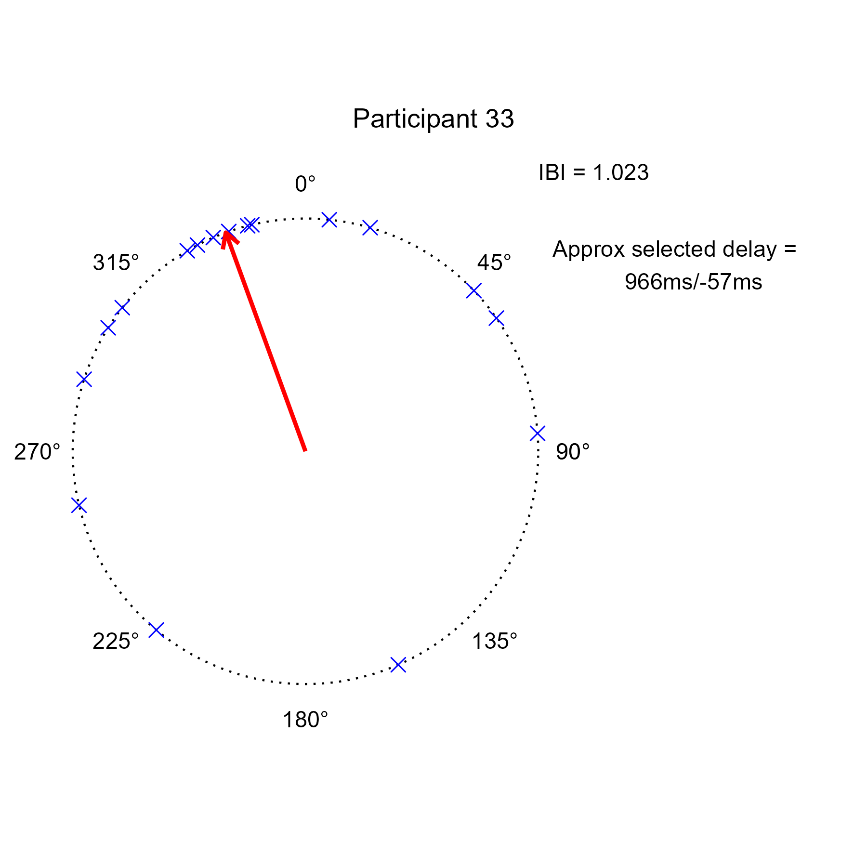

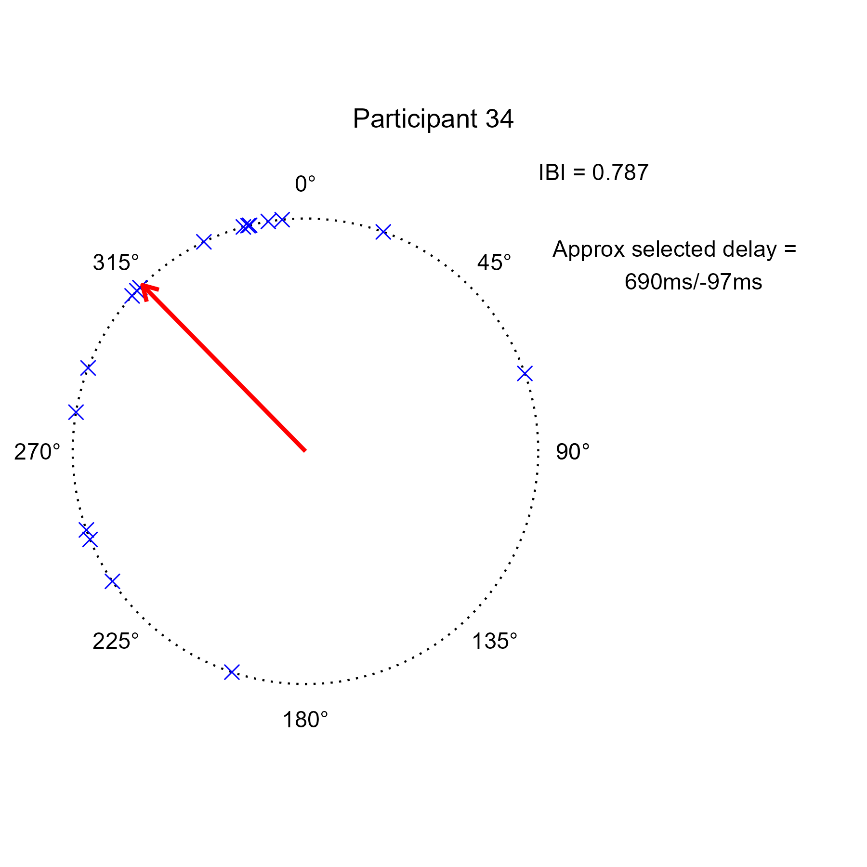

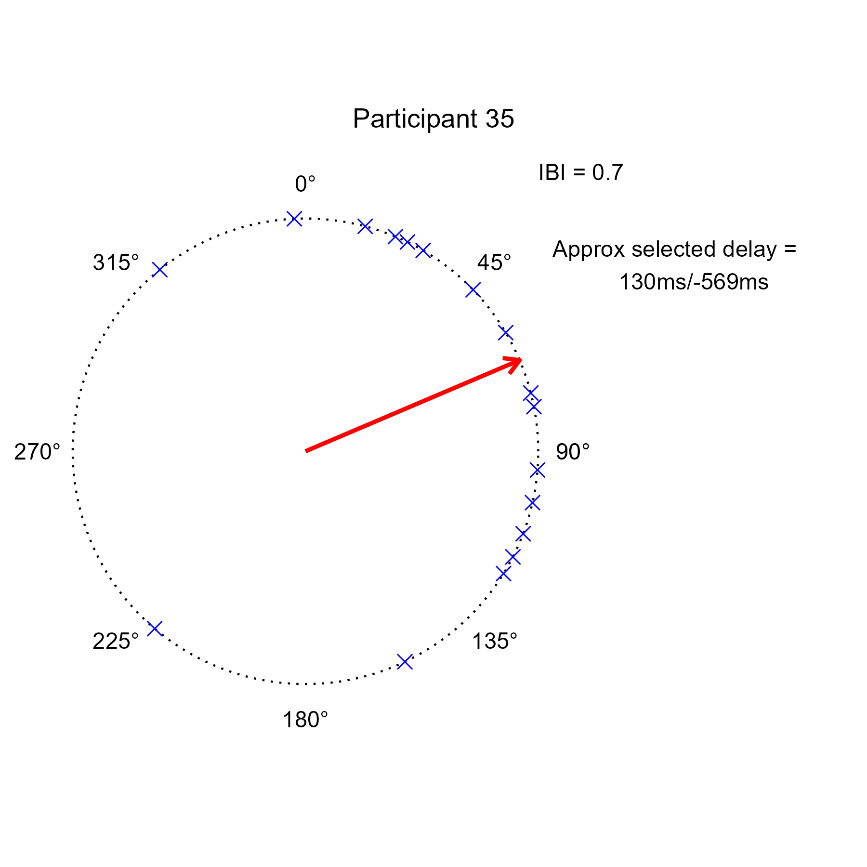

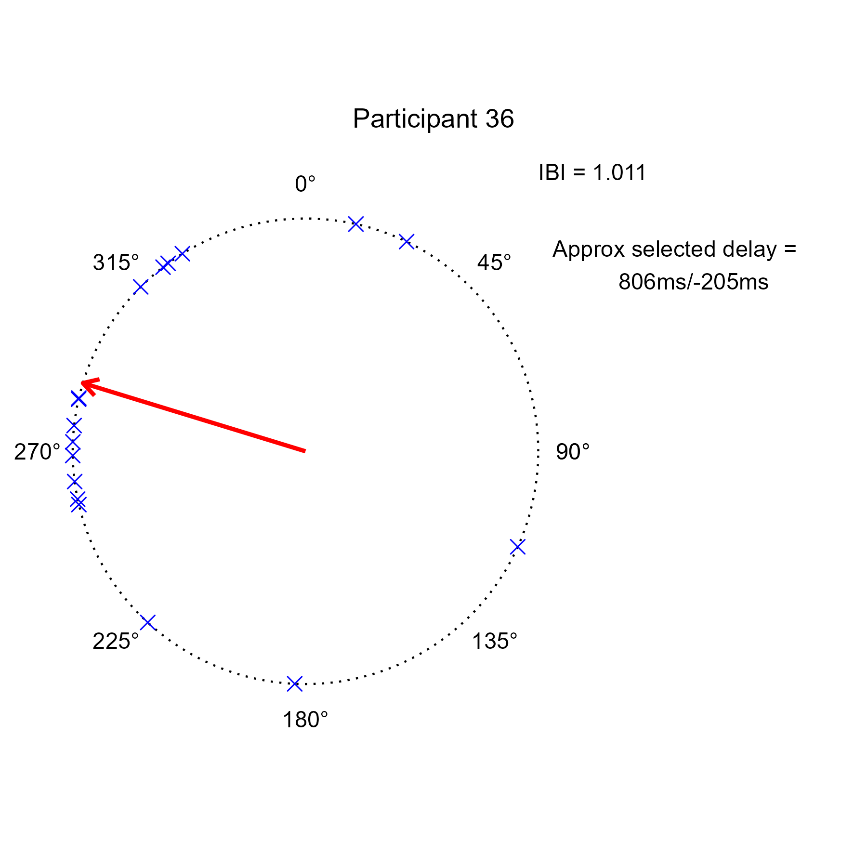

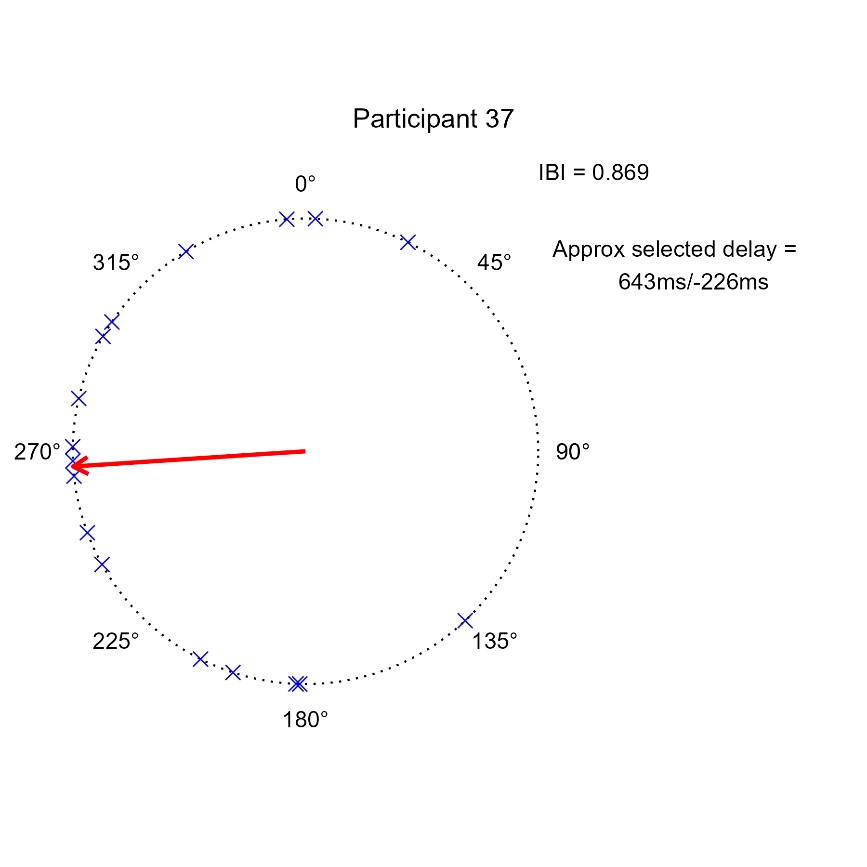

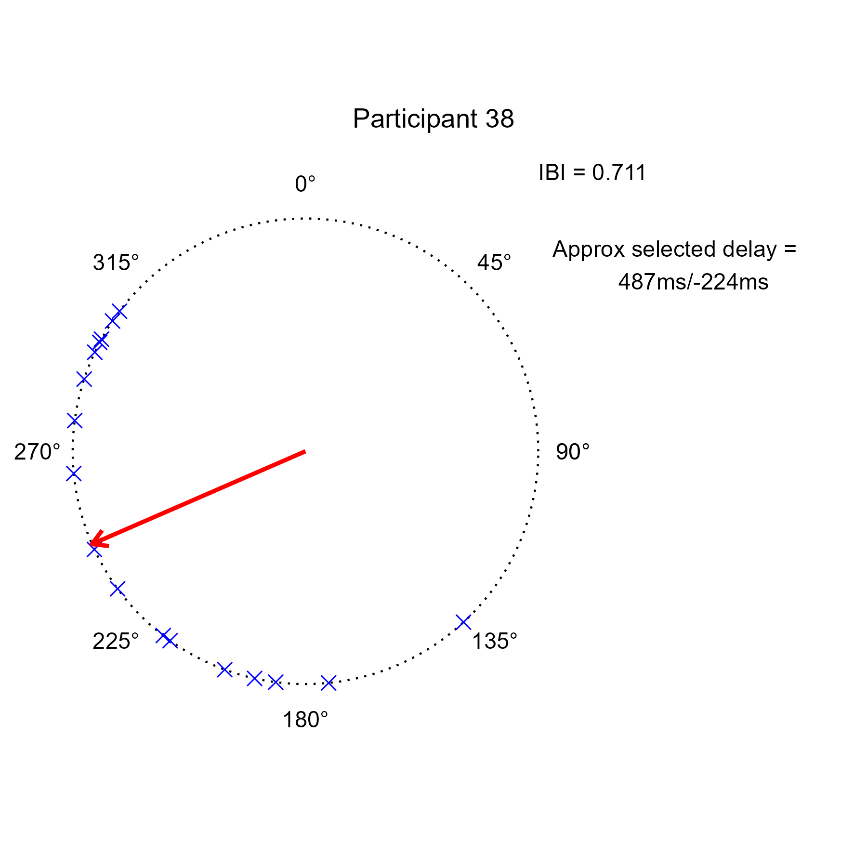

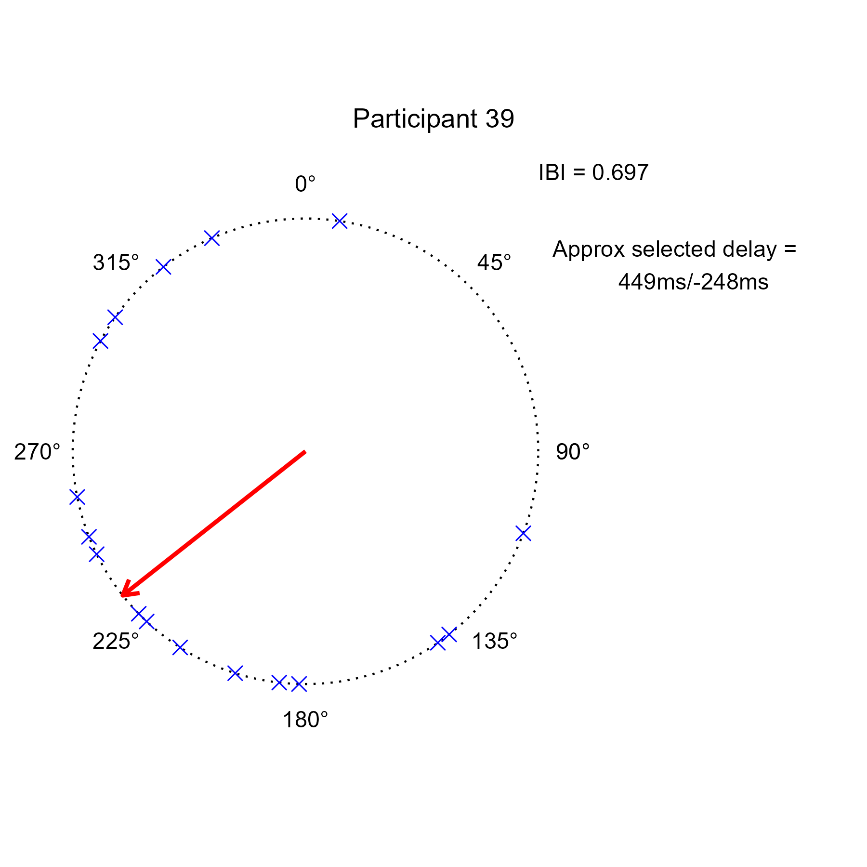

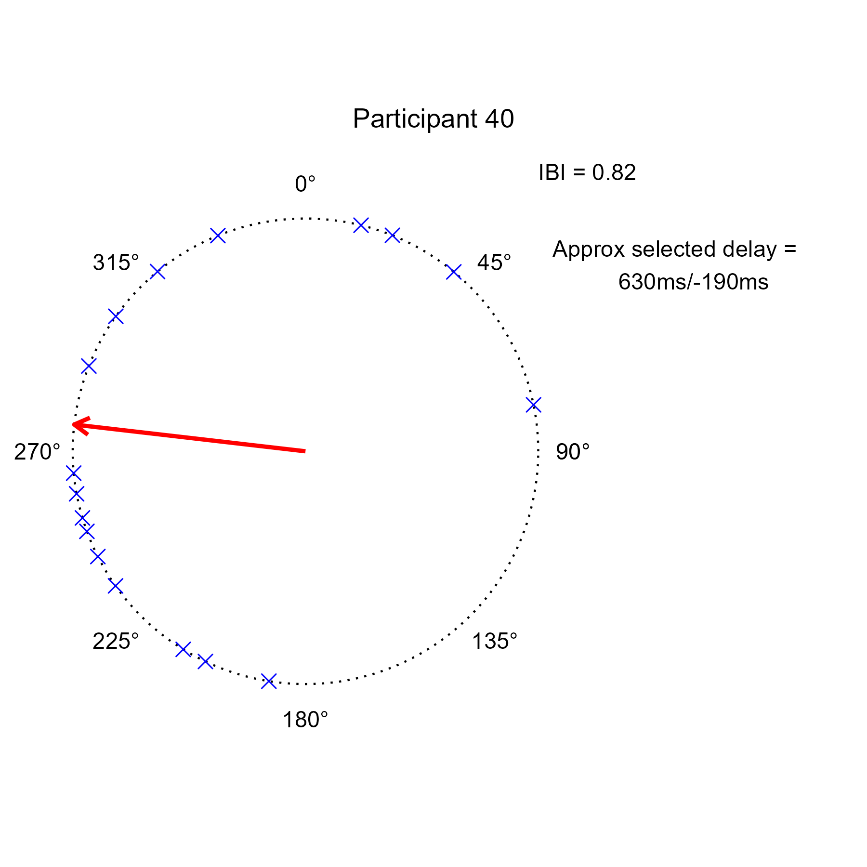

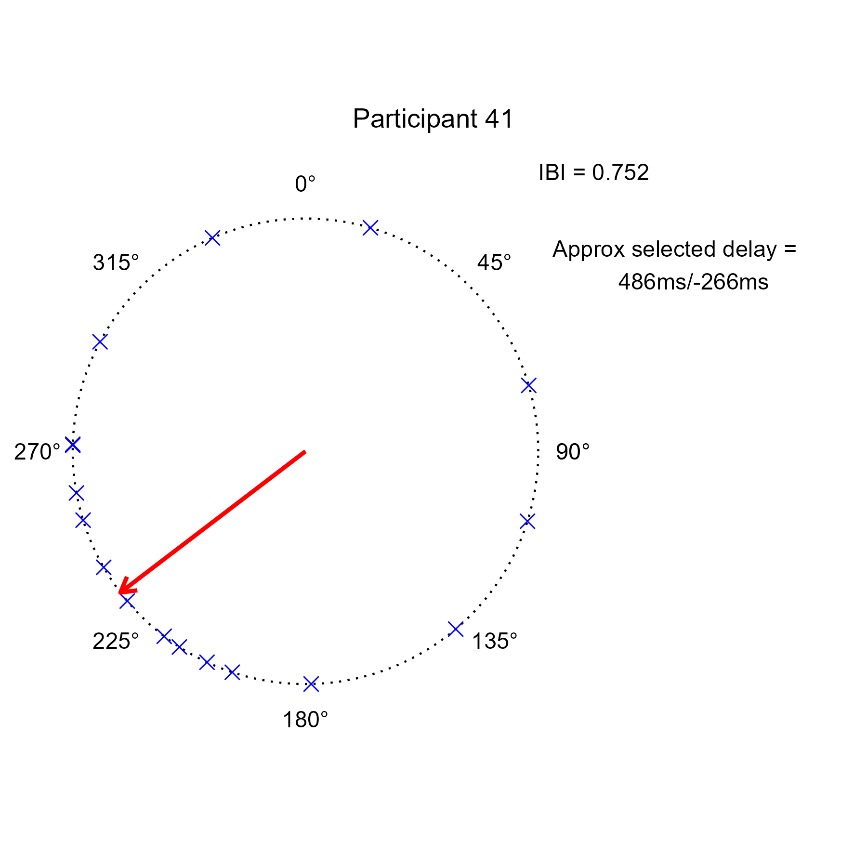

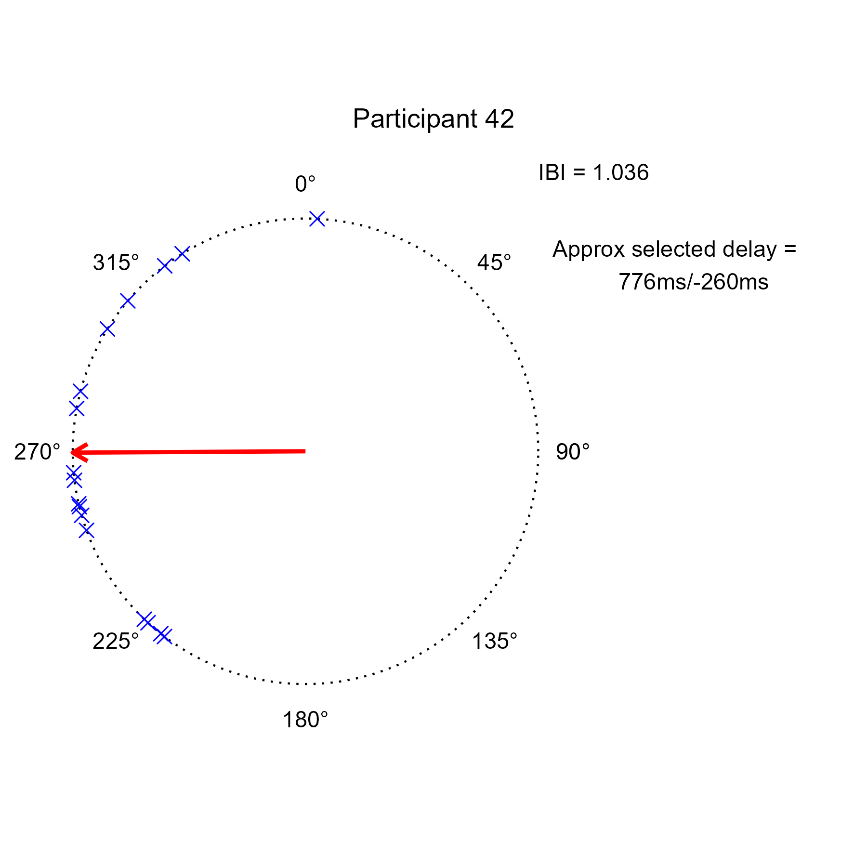
*

*
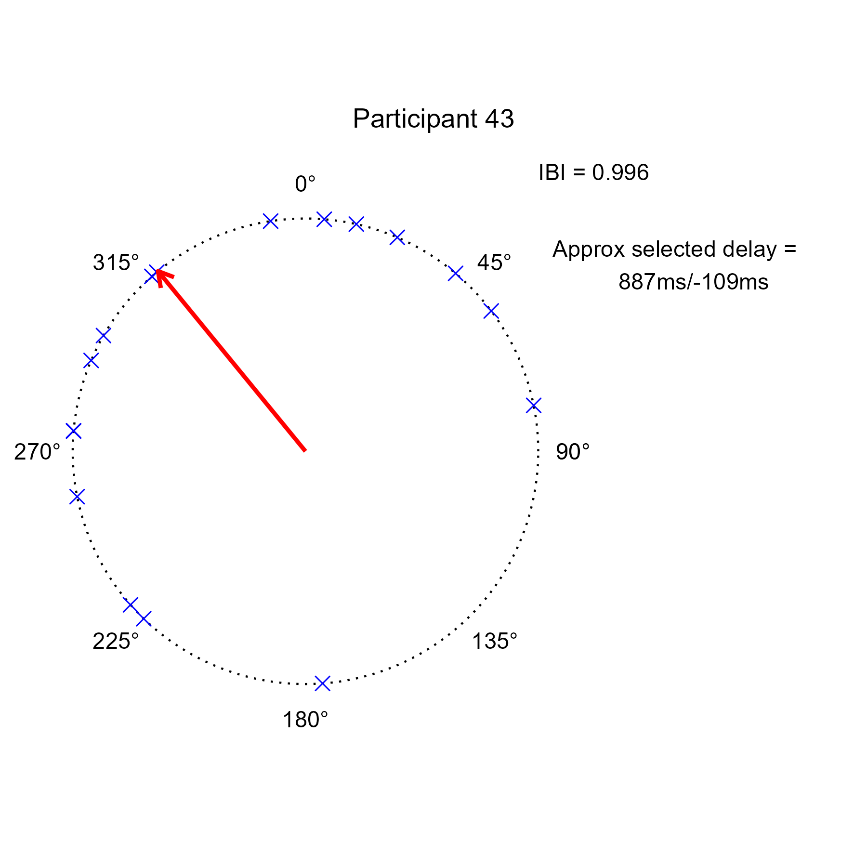
*

Supplement 6:

*The number of participants who passed the screener who selected delays aligned with the predefined synchronous and asynchronous delays used in the 2AFC-HDT*

|  | Assumed temporal precision | | |
| --- | --- | --- | --- |
|  | 50ms | 100ms | 150ms |
| Synchronous (0ms) | 0 (0%) | 5 (20.0%) | 9 (36.0%) |
| Asynchronous (300ms) | 1 (4.0%) | 3 (12.0%) | 3 (12.0%) |
| Outside of window | 24 (96.0%) | 17 (68.0%) | 13 (52.0%) |

*Note.* 25 participants both completed a screener task and passed it. For the other 18 participants, they either did not complete the screener task, or did not pass it (i.e. had too few valid trials or scores at chance). We repeated our main analysis in only those who passed the screener.
